# Predicting the Activity of Unidentified Chemicals in Complementary Bioassays from the HRMS Data to Pinpoint Potential Endocrine Disruptors

**DOI:** 10.1021/acs.jcim.3c02050

**Published:** 2024-03-25

**Authors:** Ida Rahu, Meelis Kull, Anneli Kruve

**Affiliations:** † Institute of Computer Science, University of Tartu, Narva mnt 18, Tartu 51009, Estonia; ‡ Department of Materials and Environmental Chemistry, Stockholm University, Svante Arrhenius Väg 16, Stockholm SE-106 91, Sweden; § Department of Environmental Science, Stockholm University, Svante Arrhenius Väg 16, Stockholm SE-106 91, Sweden

## Abstract

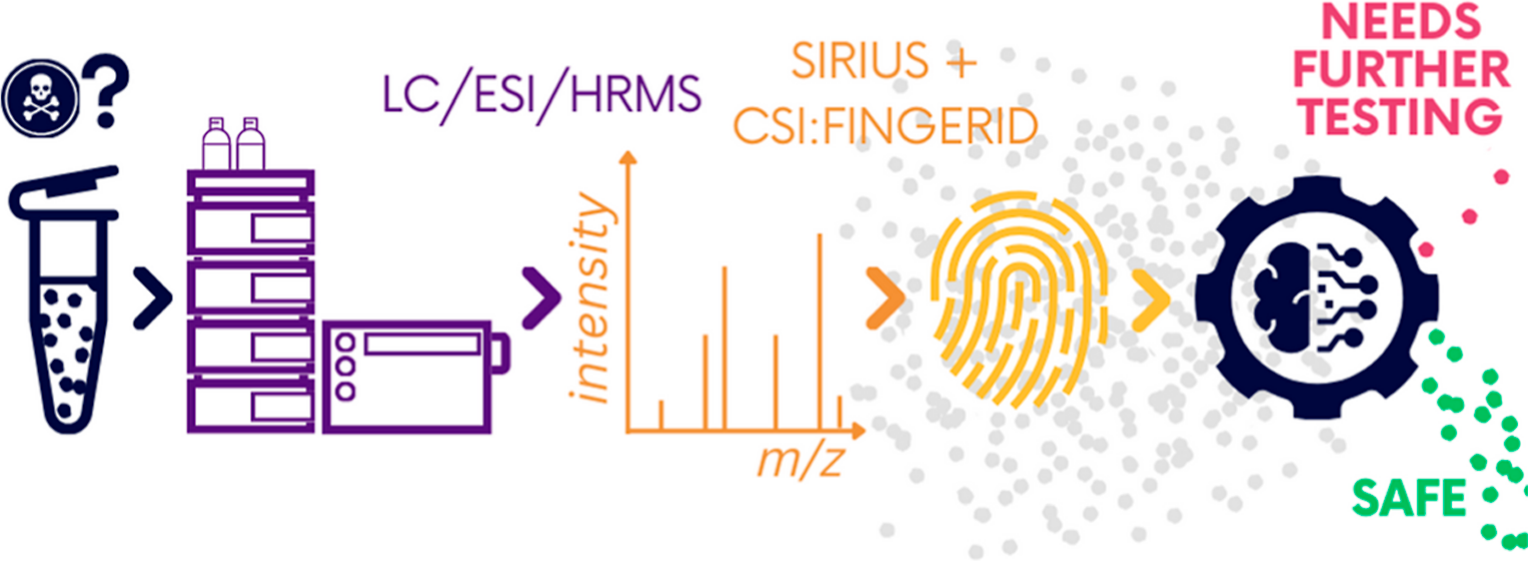

The majority of chemicals detected via nontarget liquid chromatography high-resolution mass spectrometry (HRMS) in environmental samples remain unidentified, challenging the capability of existing machine learning models to pinpoint potential endocrine disruptors (EDs). Here, we predict the activity of unidentified chemicals across 12 bioassays related to EDs within the Tox21 10K dataset. Single- and multi-output models, utilizing various machine learning algorithms and molecular fingerprint features as an input, were trained for this purpose. To evaluate the models under near real-world conditions, Monte Carlo sampling was implemented for the first time. This technique enables the use of probabilistic fingerprint features derived from the experimental HRMS data with SIRIUS+CSI:FingerID as an input for models trained on true binary fingerprint features. Depending on the bioassay, the lowest false-positive rate at 90% recall ranged from 0.251 (sr.mmp, mitochondrial membrane potential) to 0.824 (nr.ar, androgen receptor), which is consistent with the trends observed in the models’ performances submitted for the Tox21 Data Challenge. These findings underscore the informativeness of fingerprint features that can be compiled from HRMS in predicting the endocrine-disrupting activity. Moreover, an in-depth SHapley Additive exPlanations analysis unveiled the models’ ability to pinpoint structural patterns linked to the modes of action of active chemicals. Despite the superior performance of the single-output models compared to that of the multi-output models, the latter’s potential cannot be disregarded for similar tasks in the field of *in silico* toxicology. This study presents a significant advancement in identifying potentially toxic chemicals within complex mixtures without unambiguous identification and effectively reducing the workload for postprocessing by up to 75% in nontarget HRMS.

## Introduction

During their lifetime, individuals are exposed to a diverse array of chemicals from dietary intake, pharmaceuticals, household cleaning agents, environment, etc. Some of these chemicals may harm human health, and recent studies^1,2^ in medical genetics and genomics have uncovered the role of gene–environment interactions in the development of different disorders. For example, it has been found that roughly two-thirds of chronic disease risk cannot be solely attributed to genetics but instead may result from complex interactions between genes and the environment.^3^ Moreover, one in six premature deaths worldwide is reported to be caused by pollution.^4^

Traditionally, chemical pollution is often attributed to a specific group of industrial chemicals, allowing for targeted regulatory measures. However, contemporary scientific methods shed light on a more complex reality, leading to awareness that humans are exposed to a diverse mixture of chemicals. Fortunately, the advent of nontarget liquid chromatography high-resolution mass spectrometry (LC/ESI/HRMS) has enabled the simultaneous detection of numerous chemicals within real-world mixtures.^5−7^ Despite this progress, only a fraction (up to 5%) of chemicals that humans are exposed to are unequivocally identified,^7−9^ and information regarding the toxicity of identified chemicals is either insufficient or entirely absent.^10^ Therefore, significant gaps persist in the evaluation of the toxic effects associated with these detected chemicals.

In order to fill the gaps in the data, address ethical concerns related to animal testing, and overcome resource and time constraints associated with traditional toxicity testing methods, *in vitro* experiments measuring bioassay endpoints have gained popularity.^11^ Additionally, this growing pool of available toxicity data is complemented by advances in the field of *in silico* toxicology. *In silico* methods, including read-across,^12^ structural alerts,^13^ quantitative structure–activity relationship,^14^ and various machine learning models,^15,16^ have made it possible to screen a vast number of chemicals efficiently. However, these methods necessitate the availability of structural data as an input, which is frequently absent for a significant portion of chemicals in real-world samples analyzed with nontarget HRMS. Nevertheless, there is a promising workaround.

The molecule’s toxicity is often linked to specific structural patterns known as toxicophores.^17^ For instance, the presence of a hydroxyphenyl group has been associated with estrogenic and androgenic endocrine-disrupting properties.^18^ When chemical mixtures are analyzed with LC/HRMS, such structural information about unidentified substances is available: MS^2^ spectra from LC/HRMS fragmentation experiments can show the presence of specific functional groups in the molecule (e.g., hydroxyphenyl group). Extracting this information becomes feasible using tools like SIRIUS+CSI:FingerID,^19^ which map structural patterns found in spectra to molecular fingerprint features. Recently, Peets et al.^20^ demonstrated that these features could be used to predict the ecotoxicity (expressed as *in vivo* 50% lethal concentration or effective concentration) of unknown chemicals. Furthermore, Arturi and Hollender^21^ investigated the possibilities of utilizing fingerprint features for classifying unidentified chemicals based on their biochemical activity across multiple molecular endpoints.

These recent results underscore the potential of this workaround, illuminating the broader applicability of fingerprint features computable with SIRIUS+CSI:FingerID in toxicity prediction. In this work, we employ 12 bioassays from the Tox21 10K library^22^ (∼10,000 licensed drugs and environmental chemicals), which are related to endocrine disruptors (EDs), as a case study. EDs are chemicals that interfere with any aspect of hormone action in the body and thus affect the normal functioning of the endocrine system,^23^ making them a focal point for risk assessment and management plans.^24,25^ We acknowledge that specific bioassay endpoints utilized in this study may not have a direct and straightforward association with the endocrine-disrupting activity of chemicals. These connections may involve intricate mechanisms; however, for the sake of simplicity, we will refer to the observed activity in these bioassays here as indicative of the endocrine-disrupting activity. We aim to assess the suitability of fingerprint features extracted from the MS^2^ spectra for predicting the activity of chemicals across a wide range of bioassays and investigate the feasibility of utilizing the HRMS data, coupled with SIRIUS+CSI:FingerID, with the required accuracy within this approach. We hypothesize that the incorporation of Monte Carlo sampling can enhance the usage of probabilistic fingerprint features generated by SIRIUS+CSI:FingerID, serving as an input for models trained on binary fingerprint features calculated from the structural representations of chemicals in this context. Additionally, considering correlations in biochemical pathways, we hypothesize that multi-output models may outperform single-output models. Accordingly, we delve into an investigation of whether these correlations can indeed augment the predictive accuracy.

## Data and Methods

### Toxicity Data

We employed the Tox21 Data Challenge^26^ (https://tripod.nih.gov/tox21/challenge/) training set that encompasses 12 bioassays, consisting of seven nuclear receptor [activators of aryl hydrocarbon receptor (nr.ahr), androgen receptor (nr.ar), androgen receptor ligand-binding domain (nr.ar.lbd), estrogen receptor (nr.er), estrogen receptor ligand-binding domain (nr.er.lbd), peroxisome proliferator-activated receptor gamma (nr.ppar.gamma), and aromatase inhibitors (nr.aromatase)] and five stress response [activators of antioxidant response element (sr.are), heat shock response signaling pathway (sr.hse), p53 signaling pathway (sr.p53), ATPase family AAA domain-containing protein 5 (sr.atad5), and disruptors of mitochondrial membrane potential (sr.mmp)] panel pathways (Supporting Information Section S1). Each chemical, represented by its SMILES notation, was associated with activity information in the corresponding bioassay, categorized as active (″1″), inactive (″0″), or inconclusive (″NaN″).

Combining Tox21 dataset files yielded a consolidated collection of 11,764 instances: 5090 chemicals with unique SMILES and 6674 with multiple entries. The duplicate entries occasionally exhibited conflicting experimental results. For deduplication, a chemical was considered active in a specific assay if it was reported to be active in at least one corresponding experiment. After deduplication, the dataset consisted of 8043 unique SMILES notations.

Next, we processed the dataset to ensure compatibility with LC/ESI/HRMS analysis. We identified 1600 chemicals with disconnected structures (salts, coordination complexes). For these particular chemicals, we diligently worked to eliminate ions with no widely recognized endocrine-disrupting activity (e.g., Na^+^, K^+^, Ca^2+^, nitrate, acetate ions) as well as solvent molecules (e.g., H_2_O and ethanol) while neutralizing all the remaining ions, considering the valency of the atoms. To accomplish this, we used the *rdkit.Chem.SaltRemover* module and the *neutralize_atoms()*(27) function from the open-source cheminformatics software *RDKit*(28) in Python. It is important to note that we assume that the overall biochemical activity of the chemical would remain unaffected by the removal of such ions. In cases where it remained unclear which ion might potentially possess the endocrine-disrupting activity, the chemical was excluded from the dataset. We also employed expert knowledge to evaluate the suitability of each chemical for mass spectrometric analysis individually (i.e., chemicals such as indium arsenide were discarded).

**Figure 1:**
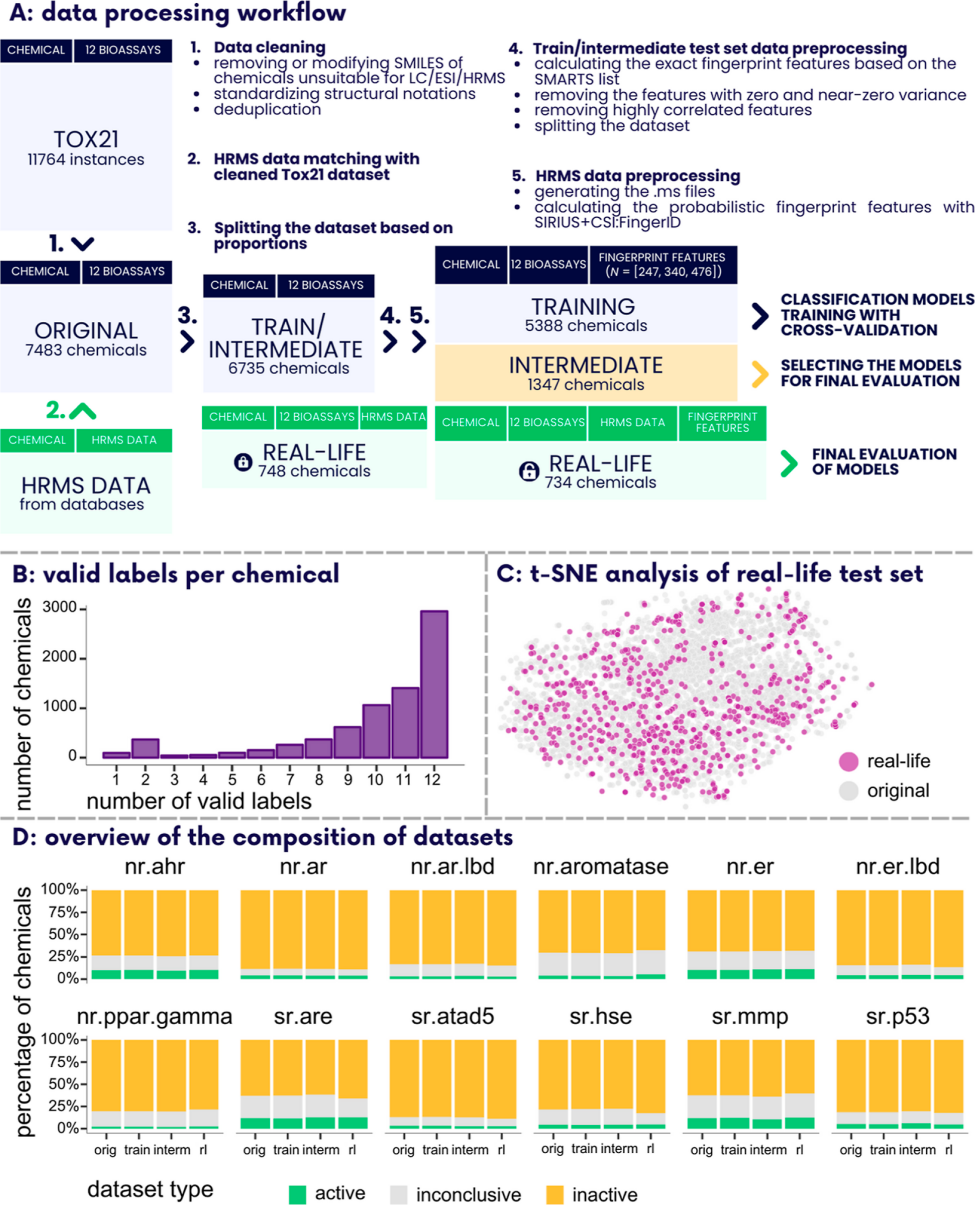
(A) Data processing workflow used in the current study. (B) Number of valid labels (active or inactive) per chemical in the original dataset. 2961 chemicals (around 40%) had labels for all the bioassays, i.e., about 60% of the chemicals had at least one missing (inconclusive) label. (C) Results of t-SNE analysis^29^ carried out by using the Python module *scikit-learn* and the function *TSNE()*.^30^ We employed the fingerprint features computed using SIRIUS+CSI:FingerID (ver. 5.6.3) to assess whether the real-life test set (highlighted in purple) effectively represents the original chemical space. (D) Proportions of the active, inactive, and inconclusive chemicals per bioassay in the original (orig) dataset (dataset obtained after deduplication and unsuitable chemicals removal from Tox21 data; 7483 chemicals), training (train) set (used for training the models; 5388 chemicals), intermediate (interm) test set (used for testing the models and selecting the final models; 1347 chemicals), and real-life (rl) test set (used for final evaluation of models; 734 chemicals). Each subplot illustrates one bioassay.

Lastly, we standardized the SMILES format of all chemicals using the *StandardizeSmiles()* function from *RDKit*. In conclusion, 7483 unique SMILES remained after data cleaning for model training and testing, and we refer to it as the original dataset (Figure 1A).

### Mass Spectrometric Data

In order to evaluate the applicability of the approach in nontarget screening, experimental HRMS data were obtained from two databases: MassBank (version 2022.06a)^31^ and MassBank of North America (MoNA).^32^ Initially, the high-resolution tandem mass spectra (MS^2^ data) were extracted from MassBank and matched with a cleaned Tox21 dataset based on standardized SMILES, yielding over 1000 chemicals with MS^2^ and toxicity data. From those chemicals, we selected 748 (10% of the original dataset) to form the so-called real-life test set (Figure 1A). Due to the data imbalances and varying levels of missing labels (Figure 1B), conventional random sampling was unfeasible for selecting a representative subset. Instead, we calculated the proportions of chemicals with active, inactive, and inconclusive labels for each bioassay within the original dataset and performed repeated random sampling until the subset matched these proportions, following a similar approach to stratified sampling (Figure 1C). For instance, the proportions of active, inactive, and inconclusive chemicals in the nr.ahr assay were approximately 10.08%, 73.54%, and 16.38%, respectively. Therefore, we sampled the data until the real-life test set contained about 75 active, 550 inactive, and 123 inconclusive chemicals in the corresponding assay. The real-life test set was exclusively reserved for the final evaluation of the selected models, and the remaining 6735 chemicals were used for model training and intermediate testing.

### Data Preparation for Training and Intermediate Testing of the Models

The absence of experimental HRMS data for all chemicals limited our ability to train the models on probabilistic molecular fingerprints generated with SIRIUS+CSI:FingerID. Therefore, the complete set of SMARTS (SMILES arbitrary target specification) corresponding to the molecular fingerprint features computed by SIRIUS+CSI:FingerID was leveraged (R package *rcdk*(33)) to compute exact fingerprint features for all chemicals based on their SMILES notation. We deliberately retained only the 3494 fingerprint features consistent across positive and negative electrospray ionization (ESI) modes in SIRIUS+CSI:FingerID. This strategic choice allows us to address the challenge posed by the limited information regarding the amenability of different chemicals in positive and negative ESI modes, information that would be necessary for training independent models for each mode. Moreover, adopting this approach ensures the versatility of classification models, making them applicable regardless of the ionization mode used in the analysis and simplifying their overall implementation.

Furthermore, the fingerprint features with zero and near-zero variance, together with highly correlated ones, were removed by utilizing functions *nearZeroVar()* (with default parameters) and *findCorrelation()* from R package *caret*.^34^ We evaluated three different cutoff values (0.7, 0.8, and 0.9) for removing correlated features, resulting in three datasets with 247, 340, and 476 fingerprint features (Figure 1A).

To partition the data into training and intermediate test sets that accurately represent the distribution of toxicity data, we employed the anticlustering algorithm^35^ using the *anticlustering()* function from the R package *anticlust*.^36^ This algorithm divides the input data into *K* heterogeneous groups that are as similar as possible to each other (anticlusters) by maximizing the clustering objective function. The approach was chosen due to the highly imbalanced nature of the original dataset and the substantial number of missing labels. To obtain the 80/20 training and intermediate test sets, we set *K* to 5: all chemicals were assigned to one of the five anticlusters, and one of them was randomly allocated as an intermediate test set.

### Data Preparation for the Final Evaluation of the Models

For each chemical within the real-life test set, we generated ″.ms″ files (https://boecker-lab.github.io/docs.sirius.github.io/io/#input) utilizing MassBank and MoNA data. If a chemical had multiple MS^2^ spectra, all acquired under identical experimental parameters (such as the same ionization mode and instrument type) but with varying collision energies, the peak lists from each spectrum were copied into a single ″.ms″ file and listed under specific collision voltages. In order to complement the MS^1^ data, isotope patterns were computed from the chemical formula with the function *isopattern()*(37) from the R package *enviPat*, compensating for the absence of experimental data in mass spectral databases. These files served as an input for SIRIUS+CSI:FingerID (version 5.6.3) to compute fingerprint features, provided as posterior probabilities, indicating the presence of specific structural patterns.

For 14 chemicals, the available fragmentation spectra did not yield sufficient information to generate characteristic fragmentation trees, and thus, the final real-life test set encompassed 734 chemicals (Figure 1A). The distribution of active, inactive, and inconclusive chemicals per toxicity assay across the training (train), intermediate (interm), and real-life (rl) test sets effectively captures the overall chemical diversity in the original (orig) dataset (Figure 1D, more details in Supporting Information Section S2).

### Modeling

In this study, we employed two concurrent modeling approaches to develop machine learning models for predicting the activity (active or inactive) of chemicals in 12 bioassays. The first approach treated each bioassay as an independent binary classification problem, resulting in separate classification models for each biochemical pathway (single-output models). In contrast, the second approach framed the task as a multilabel classification problem, training a single model to simultaneously predict the activity of chemicals across all assays (multi-output model). The training set consisted of 5388 chemicals; however, the feature sets differed between these strategies. In the first approach, we utilized all three datasets, each generated by removing highly correlated features with distinct cutoff values (0.7, 0.8, and 0.9). Although this method offered a more refined feature selection process, our analysis revealed comparable performance of single-output models trained on different feature sets across various bioassays, suggesting a minimal impact on model outcomes. Hence, for the multi-label classifiers, we exclusively employed the feature set consisting of 476 fingerprint features (cutoff = 0.9).

Our single-output models encompass a diverse range of machine learning algorithms and their different implementations: linear discriminant analysis, logistic regression (LR), naïve Bayes, *k*-nearest neighbors, support vector machine, and decision tree algorithms as well as ensemble methods such as random forest (RF), bagging, and boosting (adaptive boosting, boosted LR, gradient boosting, including stochastic gradient boosting and extreme gradient boosting). Also, neural network models were trained in this approach. All the trained models are presented in Supporting Information Section S3.

We also employed down-sampling, up-sampling [*downSample()* and *upSample()* from R package *caret*], synthetic minority oversampling^38^ [SMOTE; *smote()* from R package *performanceEstimation*], and random oversampling [ROSE; *ROSE()* from R package *ROSE*(39)] to address imbalanced data as the proportion of active chemicals ranged from 2.3% to 12.1%, depending on the assay (Supporting Information Section S5). Therefore, each binary classifier was trained for each bioassay on 15 different datasets: three different feature sets and the original imbalanced dataset together with four balanced datasets.

To develop multilabel classifiers, we utilized deep neural networks (DNNs), which demonstrated the best performance in the Tox21 Data Challenge with different architectures. These involve DNNs with up to four hidden layers using the rectified linear unit as an activation function. The number of units per hidden layer ranged from 512 to 8192, and the output layer comprised 12 sigmoid units, one for each of the 12 toxicity assays. Batch normalization was applied to address issues related to vanishing/exploding gradients. We incorporated dropout, L2 regularization, and early stopping techniques to mitigate overfitting. Due to the missing labels, the commonly used cross-entropy loss function was not applicable, and its modified version, where data points with missing labels were excluded from the loss calculation, effectively setting their loss to zero, was used. For optimization, we employed the Adam optimizer. All the hyperparameters and architectures considered are given in Supporting Information Section S4. In total, we applied 1080 different settings for multi-output model training.

We harnessed the R package *caret* for training the single-output models, and for training the multi-output models, we used the *Keras* library via *TensorFlow*(40) for R. A grid search with 10-fold cross-validation was employed to select the optimal set of hyperparameters in both strategies. While working with the original imbalanced training set, the anticlustering algorithm was used to generate the folds (function parameter *K* = 10) for cross-validation. Throughout the training, the performance of the models was assessed using the area under the receiver operating characteristic curve (ROC–AUC), sensitivity, and specificity metrics [*twoClassSummary()* function for single-output models]; however, only the ROC–AUC metric was used to select the optimal models.

### Model Selection and Final Evaluation

This study utilized the false-positive rate (FPR) at 90% recall (also known as the true-positive rate, TPR; i.e., the false-negative rate is limited to 10%) as a key metric (FPR_TPR = 0.9_) for model evaluation and selection. Following model training, we assessed each model’s performance on the intermediate test set, and the top-performing models, determined by their FPR_TPR = 0.9_, were selected for further evaluation on the real-life test set. We also recorded balanced accuracy and ROC–AUC values to facilitate comparisons with the Tox21 Data Challenge results.

The fingerprint features in the real-life test set are presented as posterior probabilities instead of binary values. Instead of a binary vector, SIRIUS+CSI:FingerID outputs a vector of probabilities based on the HRMS data where each value corresponds to one structural pattern (fingerprint feature). This value indicates the probability of the specific structural pattern being present in the chemical causing the observed HRMS data. Consequently, an additional preprocessing step is required to convert these probability vectors into binary feature vectors suitable for toxicity predictions by using the trained models. In a straightforward so-called naïve approach (for example, employed in the work of Peets et al.^20^), one might use a fixed threshold value, such as 0.5, where any predicted probability equal to or greater than 0.5 is considered as the fingerprint feature being present (denoted as ″1″), and values less than 0.5 are marked as the fingerprint feature being absent (denoted as ″0″). However, determining an appropriate threshold value can be a complex challenge.

To address this complexity, we implemented a Monte Carlo sampling strategy. For each chemical, every fingerprint feature that is used as an input in our trained models was sampled *N* times (experimentally selected *N* = 10,000) using the SIRIUS+CSI:FingerID outputted probability (*p*) that the specific feature should have a value 1 and the probability of 1 – *p* that it should have a value 0. Next, we used all the obtained *N* datasets as an input for our trained classifiers. Finally, we calculated the average of all the predictions per chemical to generate the final prediction regarding its bioactivity. We assessed the models’ performance by calculating the FPR_TPR = 0.9_ in this context.

We also utilized the SHapley Additive exPlanations (SHAP)^41^ technique via functions from the R package *ModelOriented/treeshap*(42) to provide an insight into machine learning models’ predictions.

## Results and Discussion

### Performance of the Single-Output Models on the Intermediate Test Set

In this study, we developed machine learning models to predict the endocrine-disrupting activity of chemicals based on structural information obtained from HRMS analysis. More precisely, we employed molecular properties extracted from SIRIUS+CSI:FingerID to accurately classify chemicals as either active or inactive across 12 bioassays within the Tox21 Data Challenge dataset.

In the context of discovering EDs based on the data obtained in HRMS analysis, the models must possess a high recall, effectively detecting the majority of EDs present within the analyzed sample. Simultaneously, minimizing the number of chemicals incorrectly classified as active (low FPR) is required to manage the postprocessing workload. As a result, we employed FPR_TPR = 0.9_ as a criterion for model evaluation and selection.

**Figure 2:**
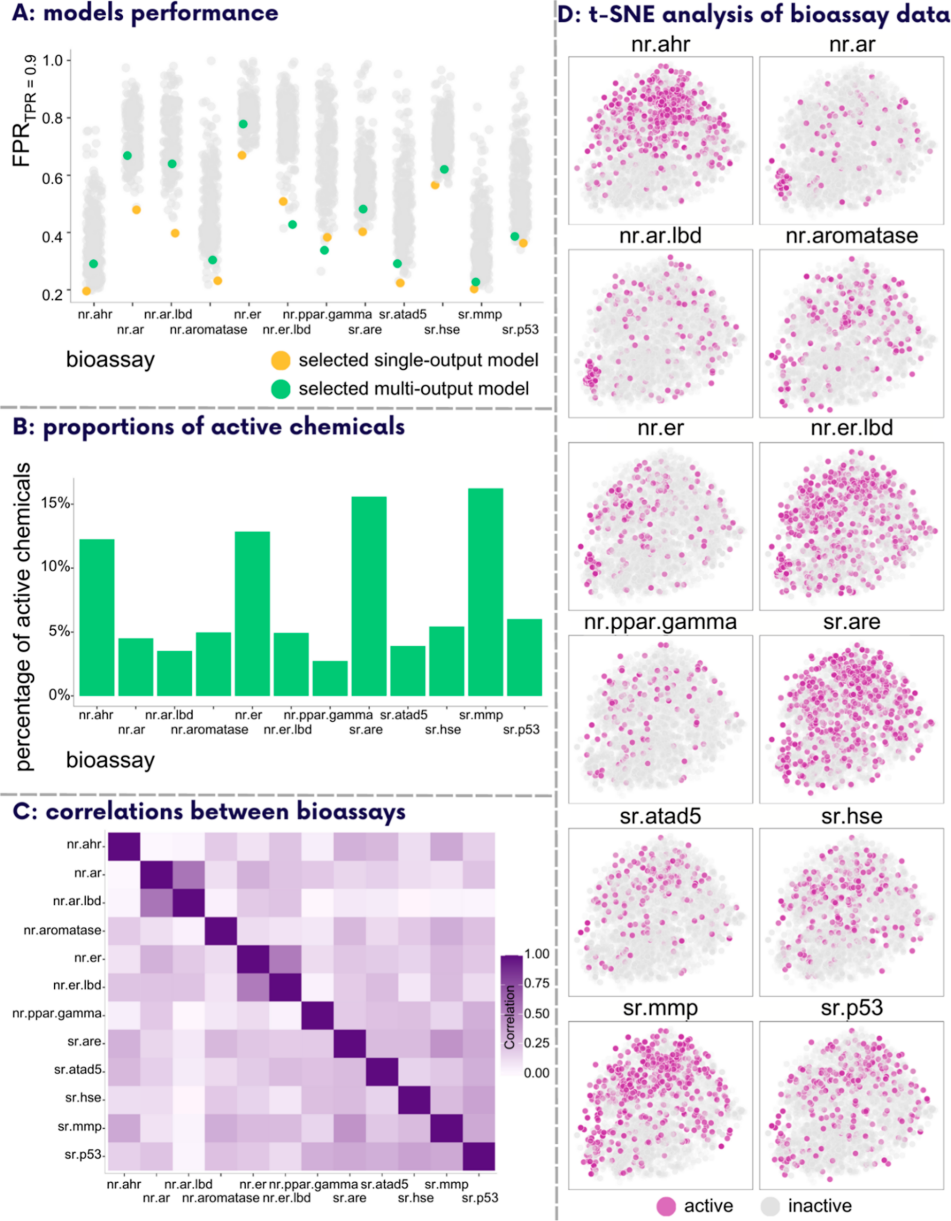
(A) Models’ performance on the intermediate test set expressed as FPR_TPR = 0.9_ (metric employed to select the models for final evaluation). Trained models with a metric value of 1 were excluded from the comparison. The data points highlighted in yellow represent the single-output models selected for final evaluation on the real-life test set, while the data points highlighted in green represent the multi-output model chosen for the same purpose. (B) Proportions of the active chemicals per bioassay. In the calculations, only the active and inactive chemicals were considered (i.e., chemicals with inconclusive labels were discarded). (C) Pairwise correlation matrix for all of the biochemical assays. (D) Results of t-SNE analysis. Only the active (purple) and inactive (gray) chemicals are shown in every assay, and each subplot’s *x*- and *y*-axes are scaled into the same ranges for consistency and direct comparability.

The lowest achieved FPR_TPR = 0.9_ varied between 0.196 (nr.ahr) and 0.670 (nr.er) for single-output models depending on the biochemical assay (Figure 2A, models’ details in Supporting Information Section S5), implying a potential reduction of up to 80% in the postprocessing workload for nontarget HRMS. This reduction is facilitated by enabling the focus to be solely on chemicals labeled as positive (″toxic″), eliminating the need for manual interrogation and testing of all the chemicals. Additionally, among all of the algorithms utilized while training the single-output models, the final selection comprised only ensemble-based algorithms, such as RF and boosting. Based on these results, it can be concluded that the fingerprint features computable by SIRIUS+CSI:FingerID from HRMS are characteristic enough to predict the endocrine-disrupting activity of chemicals.

### Comparative Analysis of Trained Models and Tox21 Data Challenge Submissions: Unveiling Parallel Performance Trends

We noted varying levels of difficulty in modeling the activity of chemicals across different bioassays (Figure 2A). For instance, models performed exceptionally well in bioassays such as nr.ahr and sr.mmp, where the lowest FPR_TPR = 0.9_ among all models was 0.196 and 0.203, respectively. Conversely, predicting the activity of chemicals in the nr.er bioassay presented greater challenges, with the best FPR_TPR = 0.9_ being 0.670. We examined whether these fluctuations in model outcomes stem solely from the experimental design, e.g., selection of the fingerprint features or data cleaning, by comparing their performance with models submitted in the Tox21 Data Challenge.

In the Tox21 Data Challenge, the models’ performance was assessed using balanced accuracy and ROC–AUC values. We calculated the same metrics for all our trained models using the intermediate test set (Supporting Information Section S6), with the understanding that a direct numerical comparison is unfeasible due to differences in test sets; however, the general trends agree. Besides, one should keep in mind that unlike the models in the Tox21 Data Challenge,^26^ which leveraged multiple descriptor types sourced from structural information and external data from the literature, our models were constrained to utilizing solely fingerprint features computable from HRMS with SIRIUS+CSI:FingerID.

In both the Tox21 Data Challenge and our study, the bioassays nr.ahr and sr.mmp appeared to have the highest-performing models. In the competition, the average ROC–AUC scores for these assays exceeded 0.8; in our study, the corresponding scores were 0.856 and 0.870, respectively. The lowest average ROC–AUC scores (approximately 0.7) were observed in the competition for models designed to predict the activity of chemicals in nr.ar. and nr.ar.lbd bioassays. In the Tox21 Data Challenge, the lowest ROC–AUC among the winning models was 0.810 for predicting the activity of chemicals in the nr.er bioassay. Similarly, in the current study, the nr.er bioassay exhibited models with the lowest maximum ROC–AUC score, reaching 0.768.

Additionally, the highest balanced accuracies for Tox21 Data Challenge models ranged from 0.650 (nr.ar.lbd) to 0.904 (sr.mmp), with the lowest balanced accuracy among the winning models at 0.550 for the nr.er.lbd bioassay. Among the models trained here, the balanced accuracies of the best-performing ones ranged from 0.709 (nr.er) to 0.848 (nr.ahr). The most significant discrepancy between our study and the Tox21 Data Challenge was observed in the case of the sr.hse bioassay. While it exhibited a relatively high predictive performance in the competition (the average ROC–AUC score was approximately 0.75, and the highest ROC–AUC score was about 0.9), it resulted in the lowest average ROC–AUC score in our study (0.704).

It is evident that while the experimental design unquestionably influences model outcomes, certain assay-specific factors also exert an influence. The Tox21 Data Challenge organizers^26^ noted that assays with a higher proportion of active chemicals tended to yield a higher model performance. For instance, the consistently high performance of models developed for bioassays such as nr.ahr and sr.mmp can be attributed to a relatively high proportion of active chemicals (Figure 2B). Nevertheless, the challenges faced while modeling the activity of chemicals in nr.er (∼13% active chemicals) bioassay imply the presence of unidentified factors that also affect the modeling process, where the complexity of the biochemical pathway may have a pivotal role. Additionally, the organizers emphasized that despite the availability of several computational approaches to address data imbalance, their effectiveness is constrained by the limited real structural information that can be extracted. This limitation is consistent with our experiments, as none of the sampling techniques we employed significantly improved the results across all the bioassays. To offer insights into the quality of captured toxicophores, we conducted t-distributed stochastic neighbor embedding (t-SNE) analysis (Figure 2D).

Despite the limitations, the t-SNE plot revealed a clear separation between active and inactive chemicals for the nr.ahr assay, suggesting that fingerprint features can effectively capture discriminative information relevant to this assay. Conversely, for the nr.er bioassay, the chemicals exhibited a significantly broader dispersion on the t-SNE plot, indicating greater complexity, which may explain the lower performance for the nr.er assay.

### Correlations between Bioassays and Performance of Multi-Output Models

The t-SNE plots and pairwise correlation matrix (Figure 2C,D) effectively demonstrated similarities of specific bioassays, e.g., nr.ar.lbd and nr.ar, which is expected given their relevance to the androgen receptor signaling pathway. However, correlation analysis also reveals the complexity of these relationships. For example, chemicals acting as agonists of the antioxidant-responsive element also tend to disrupt the mitochondrial membrane potential (MMP), which is consistent with findings in the literature^43^ that demonstrate a direct link between antioxidative stress and MMP. These correlations aid in understanding why models for certain assays perform similarly and utilize similar fingerprint features. Additionally, they underscore the potential benefits of employing multi-output models in the field of *in silico* toxicology. By considering multiple bioassays simultaneously, multi-output models could leverage these correlations to extract more information from the data, potentially leading to more robust and accurate predictions.

FPR_TPR = 0.9_ of the multi-output model on the intermediate test set (ranging from 0.218 for nr.aromatase to 0.713 for nr.er bioassay) does not conclusively support the hypothesis, as in most bioassays, the selected single-output models exhibited a lower FPR_TPR = 0.9_ compared to that of the chosen multi-output model (Figure 2A; model’s details in Supporting Information Section S5). However, this variation in performance may arise from differences in the selection process. Single-output models were chosen based on their performance in predicting the endocrine-disrupting activity of specific bioassays. In contrast, for the multi-output model, the selection was made by considering the average FPR_TPR = 0.9_ across all bioassays, with the choice being determined by the lowest average value of 0.455.

To gain a deeper understanding and enable a more direct comparison of results, we expanded our analysis to incorporate single-output DNNs. These models, sharing the same architecture as the selected multi-output model, were specifically designed to predict the activity of chemicals in the nr.aromatase and nr.er biochemical pathways. Slightly higher FPR_TPR = 0.9_ values for single-output DNNs (0.227 for nr.aromatase and 0.784 for nr.er bioassay) were observed compared to that for the multi-output model (0.218 for nr.aromatase and 0.713 for nr.er bioassay); however, significant superiority of multi-output models over their single-output counterparts cannot be claimed.

Finally, it is crucial to emphasize that the observed trends in the performances of single- and multi-output models exhibit alignment across bioassays (Figure 2A). Consequently, the trend analysis conducted in the previous section with single-output models can be inferred to apply to the multi-output model as well. This consistency reinforces the robustness of our findings and ensures a coherent interpretation of the results.

### Feature Importance with SHAP Analysis

**Figure 3:**
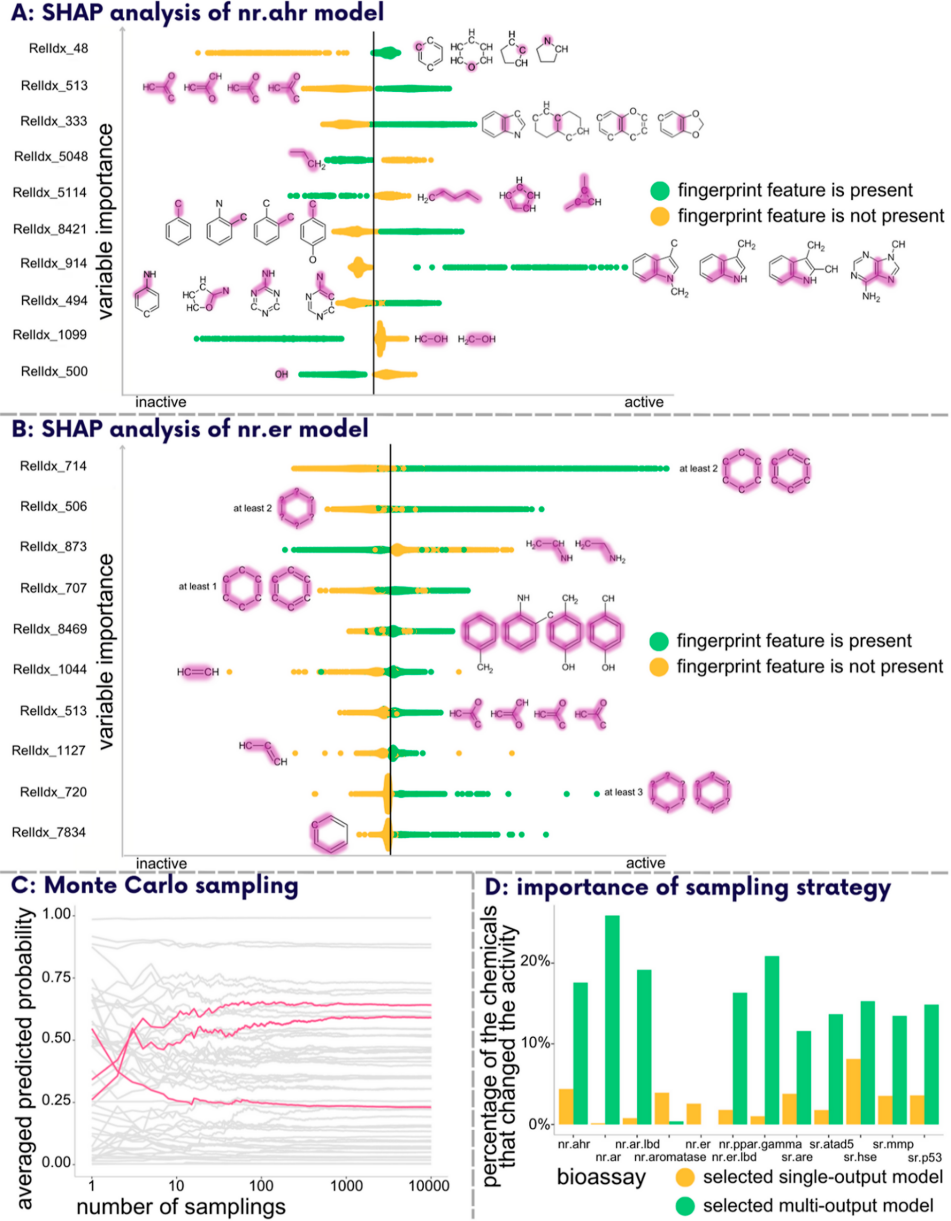
(A,B) Variable importance analysis (A) for an extreme gradient boosting classifier for nr.ahr and (B) for an RF classifier for nr.er. According to SHAP analysis, the results of the ten most important variables are shown together with their graphical descriptions from SIRIUS+CSI:FingerID, where the purple highlight indicates the exact structural patterns corresponding to the feature under investigation. The *x*-axis of the plot represents the directionality of the effect of variables. The color of the points indicates the absence (″0″) or presence (″1″) of the respective structural fragment. ″RelIdx″ refers to the absolute index system in SIRIUS+CSI:FingerID. (C,D) Comparison of the Monte Carlo sampling strategy and naïve approach for endocrine-disrupting activity prediction using single- and multi-output models. (C) Effect of different sampling iterations on the predictions of the single-output model for 50 chemicals in the nr.ahr bioassay. Each line represents the predictions made for one chemical, and the pink lines indicate the importance of adequate sampling iterations. (D) Percentage of chemicals whose endocrine-disrupting activity prediction would have been different if the naïve approach had been used instead of the optimal sampling strategy for each bioassay and model.

In order to investigate whether the final single-output models trained to predict the activity of chemicals in nr.ahr and nr.er bioassays capture meaningful toxicophores, we conducted SHAP analysis (Figure 3A,B). For the nr.ahr model, the fingerprint feature with the highest importance score (top 1 feature) is RelIdx_48, corresponding to the different ring structures. Based on the results, its presence indicates a higher likelihood of chemicals being active in the nr.ahr bioassay, which is in line with the established modes of action (MOAs) of EDs.^44^ Nevertheless, the informativeness of this feature is limited due to its broad chemical space coverage. On the other hand, the top 3 feature, RelIdx_333 (annelated rings), and the top 7 feature, RelIdx_914 (indole moiety), are highly relevant because polycyclic aromatic hydrocarbons^45^ and indole-derived chemicals^46^ are well-documented aryl hydrocarbon receptor ligands. Consequently, the most important fingerprint features capture chemical information that is consistent with the MOAs of EDs.

In the case of the nr.er model, the fingerprint feature RelIdx_714, corresponding to the structural patterns that contain at least two saturated or aromatic carbon-only rings with six atoms, received the highest importance score. This aligns with the established MOAs of EDs, emphasizing the relevance of ring systems in binding to the estrogen receptor. However, this feature encompasses a broad range of chemicals and offers limited specificity. In contrast to the nr.ahr model, where specific toxicophores were identified among the top ten important variables, none of the top ten identified features can be considered truly specific toxicophores for nr.er. While they all demonstrate a reasonable association with the MOAs, their individual contributions to the activity of the chemical in the nr.er bioassay are inconclusive.

Additionally, based on these SHAP results, it is evident that despite our efforts to eliminate highly correlated fingerprint features, some still exhibit significant chemical overlap (e.g., RelIdx_714, RelIdx_506, RelIdx_707, and RelIdx_720) and can significantly impact the robustness and performance of the models because their predictions may rely on only a small fraction of highly similar fingerprint features. We extended the SHAP analysis to gain insights into how the composition of the set of fingerprint features in the training set influences the features utilized by the models. This extension encompassed models that shared the same algorithm and were trained using an identical training set (same sampling strategy) as the final model for both nr.ahr and nr.er but contained a different number of features due to different cutoff values used in data preprocessing (Supporting Information Section S7). This analysis revealed that among the 20 most important fingerprint features extracted for each nr.ahr model, the list consistently included 4 specific features (RelIdx_333, RelIdx_505, RelIdx_513, and RelIdx_8421). Likewise, examining the 20 most important variables in the nr.er models revealed that nine of these features were shared across all three models obtained while using three distinct cutoff values (0.7, 0.8, and 0.9) to remove highly correlated features.

We also delved into how the most significant fingerprint features differed across models that utilized various algorithms. To accomplish this, we conducted SHAP analysis using models trained on the same set of chemicals and features as the model selected for the final evaluation in both assays (Supporting Information Section S7). From this assortment of models suitable for our analysis, we opted for the one with the lowest FPR_TPR = 0.9_ as a representative example. For both bioassays, the models selected in this manner utilized extreme gradient boosting and RF algorithms. In the case of nr.ahr models, 10 out of the 20 most important fingerprint features were consistent across both models (extreme gradient boosting and RF classifiers), while for nr.er models, it was 8.

The results of all the SHAP analyses indicate that, at least for some of the bioassays, fingerprint features used for training the models contain characteristic toxicophores, and machine learning models are able to learn them. This finding holds great significance, as it opens avenues for a more profound exploration of the toxicological impacts of chemicals using HRMS.

### Evaluation of Models on the Real-Life Test Set

In order to evaluate the performance of the models under near real-world conditions (utilizing a real-life test set), the fingerprint features were calculated from the HRMS data with SIRIUS+CSI:FingerID. Conversely, to fingerprint features derived from molecular structures (binary values), those generated from the HRMS data are characterized as posterior probabilities (*p*). In a straightforward (naïve) approach for converting probabilistic fingerprint features to fingerprints expressed as ″1″ or ″0″, one could assign any fingerprint feature with *p* ≥ 0.5 as present (″1″) and <0.5 as absent (″0″).^20,21^ However, determining an appropriate threshold value is challenging. Therefore, we embraced a probabilistic methodology that we refer to as a Monte Carlo sampling strategy. *N* binary feature vectors were constructed for each chemical by iteratively sampling each fingerprint feature according to its predicted probability from SIRIUS+CSI:FingerID. Consequently, this approach yielded slight variations among the *N* binary feature vectors. Each feature vector was then processed through the same pretrained model to produce a prediction. The final prediction for a chemical was determined by averaging these *N* outputs.

We observed that cumulative single-output model predictions change significantly with the increase of *N*, and for some chemicals, applying a threshold value of 0.5 to convert the model’s predicted probability into the class label would yield different outcomes when using a lower *N* compared to a higher *N*, such as 10,000. In the case of the nr.ahr bioassay, 61 out of 614 chemicals would exhibit such a behavior. Our experiments revealed that 10,000 samplings sufficed for all models, as average predictions remained constant even with increasing *N* (Figure 3C, Supporting Information Section S8).

We also conducted a comparative analysis of our Monte Carlo strategy and the previously mentioned naïve approach, with a threshold of 0.5. In the case of single-output models, less than 10% of chemicals yielded disagreeing results (Figure 3D). At the same time, for the multi-output model, the disagreement reached up to 20% for the nr.ar assay. These variations align with the characteristics of multi-output models, which simultaneously predict multiple properties of chemicals and rely on the correlation between bioassays, enabling them to learn hierarchical data representations across various tasks. Therefore, alterations in the input data also affect multiple prediction outcomes simultaneously. Consequently, the notable contrast in disagreement rates between the two model types indicates that their predictions are based on different fingerprint features and underscores the property of multi-output models to be influenced by associations in fingerprint features that might have limited effect in single-output models due to data sparsity. The results, particularly for the multi-output model, validate the effectiveness of the Monte Carlo sampling strategy in achieving consistent and reliable predictions when utilizing probabilistic SIRIUS+CSI:FingerID fingerprint features in our modeling approach.

The performance of the models on a real-life test set, quantified as FPR_TPR = 0.9_, ranged from 0.251 (sr.mmp) to 0.850 (nr.er) (Table 1). This suggests their capability to potentially reduce the postprocessing workload for nontarget HRMS analysis by up to 75% (for FPR of 0.25). Furthermore, the performance of the models on the real-life test set mirrored the patterns observed in the intermediate test set. Predicting the activity of chemicals in the nr.er bioassays remains one of the most challenging tasks, whereas both single- and multi-output models demonstrate a high performance in the sr.mmp bioassay. (The comparative results, obtained using a naïve approach with a threshold of 0.5 to utilize the probabilistic fingerprint features, are presented in Supporting Information Section S8.)

**Table 1:** Performance of the Models on the Real-Life Test Set^a^

bioassay	FPR_TPR = 0.9_	
	single-output models	multi-output models
nr.ahr	**0.408**	0.430
nr.ar.lbd	**0.688**	0.789
nr.ar	**0.824**	0.904
nr.aromatase	0.520	**0.379**
nr.er.lbd	0.844	**0.576**
nr.er	**0.850**	0.882
nr.pppar.gamma	0.570	**0.537**
sr.are	**0.690**	0.700
sr.atad5	0.856	**0.422**
sr.hse	0.795	**0.754**
sr.mmp	**0.251**	0.339
sr.p53	0.461	**0.254**

a Values are calculated using the Monte Carlo sampling strategy developed in this study (*N* = 10,000). The lowest FPR_TPR = 0.9_ value per bioassay is highlighted in bold.

Some noteworthy differences exist between the results obtained using the intermediate and real-life test sets. The single-output models consistently tend to achieve a lower FPR_TPR = 0.9_ when evaluated on the intermediate test set, with only two exceptions where the multi-output model outperforms. In contrast, both the single- and multi-output models had a lower FPR_TPR = 0.9_ in half of the bioassays on the real-life test set (Table 1). These findings substantiate the feasibility of utilizing fingerprint features derived from the HRMS data via SIRIUS+CSI:FingerID to predict chemicals’ endocrine-disrupting activity and that multi-output models hold promise for similar tasks, although additional strategies are warranted.

### Limitations and Perspectives of the Proposed Approach

This study aims to identify potentially toxic chemicals detected in HRMS and warrants further examination by leveraging fingerprint features predicted with SIRIUS+CSI:FingerID. While this approach offers advantages such as predicting the activity of unidentified chemicals in complementary bioassays, it still possesses many limitations.

For instance, although the present study utilized the Tox21 Data Challenge dataset, which is widely used in the field of *in silico* toxicology and contains a diverse set of chemicals, it might not cover the full spectrum of chemical classes found in real-world samples. Consequently, the generalization capability of the trained models could be constrained, particularly when chemical mixtures containing chemically underrepresented or unrepresented chemical classes.

Additionally, in the realm of machine learning for toxicology, a fundamental aspect is identifying and selecting appropriate features (structural patterns or structural alerts) to characterize potentially toxic chemicals effectively. In this study, the fingerprint features were predefined by the ones extractable from the HRMS data with SIRIUS+CSI:FingerID (section “Data Preparation for Training and Intermediate Testing of the Models”), and we operated under the assumption that these fingerprint features can adequately encapsulate the bioactivity of chemicals. Nevertheless, this may lead to biased predictions for some chemicals, and exploring additional information extractable from the HRMS data remains an essential research direction.

Furthermore, the high posterior probabilities (0.99) of fingerprint features derived from the HRMS data with SIRIUS+CSI:FingerID do not necessarily confirm the presence of that fingerprint feature. Even under an assumption that the overall accuracy of SIRIUS+CSI:FingerID is as high as 99% for all of the fingerprint features, it implies that approximately 35 out of 3494 overlapping fingerprint features used in this study may still harbor inaccuracies and might have a decisive effect if occurring for high-importance features (Supporting Information Section S9). In most instances, the molecular properties predicted by SIRIUS+CSI:FingerID align well with those calculated from the SMILES notations (true fingerprint features). Our simplified analysis, considering posterior probabilities ≥0.5 as the presence of fingerprint feature, showed that most features exhibited mispredictions for less than 4% of the chemicals (Supporting Information Section S9). However, for the predictions for the benzeneamine group, the SIRIUS+CSI:FingerID and structure-based disagreed for >12% of the chemicals. Aniline and its derivatives are known chemicals that can impact steroidogenesis, potentially making the benzeneamine group a crucial toxicophore.^47^ This example highlights that if fingerprint features are complicated to predict from MS^2^ spectra, it could result in misclassifications in activity toward bioassays and should be taken into account when implementing this approach.

Furthermore, we noted that certain chemicals exhibited more frequent deviations in their predicted fingerprint features compared to that of the true ones (Supporting Information Section S9). This phenomenon could be attributed to a lack of meaningful fragments in their MS^2^ spectra, which hinders the construction of comprehensive fragmentation trees and subsequently impacts the prediction of molecular properties. This limitation was also evident in the present study when utilizing the HRMS data to calculate fingerprint features for a real-life test set, where fragmentation trees could not be calculated for 14 chemicals (section “Data Preparation for the Final Evaluation of the Models”). This observation emphasizes the vital importance of high-quality experimental data, particularly data-rich fragmentation spectra. It underscores the necessity for experimental methodologies capable of assessing spectral quality on the go, enabling the adjustment of collision energies to enhance data quality.

## Conclusions

The study utilized the HRMS data from nontarget LC/HRMS analysis to predict the endocrine-disrupting activity of chemicals in multiple bioassays prior to their unequivocal structural identification. We observed that probabilistic fingerprint features computed from the HRMS data with SIRIUS+CSI:FingerID exhibit sufficient characteristics, allowing for a 90% recall while maintaining a low FPR (ranging from 0.251 to 0.850) in predicting the activity of chemicals in 12 bioassays in the Tox21 10K library. The classifiers revealed characteristic structural patterns of toxic chemicals associated with their MOAs. Additionally, our experiments showed that the Monte Carlo sampling-based technique of the probabilistic fingerprint features is essential for consistent activity predictions from the HRMS data. While suggesting the advantages of predicting the activity of chemicals in multiple bioassays simultaneously, the study highlights the need for further experiments and analysis to validate the proposed methodology’s performance and selection strategies. Overall, this research marks a significant advancement in identifying toxic chemicals in complex mixtures without chemical identification, providing a strong foundation for future studies and the development of novel approaches to address knowledge gaps in evaluating their toxic effects downstream.

## Data Availability

The code and data used for this work are publicly available at https://github.com/kruvelab/MS2Tox/development/Tox21.

## Supporting Information Available

Prediction of the activity of unidentified chemicals in complementary bioassays from the HRMS data to pinpoint potential EDs (PDF)
