# Supplementary material for: Predicting the Activity of Unidentified Chemicals in Complementary Bioassays from the HRMS Data to Pinpoint Potential Endocrine Disruptors
Source: J Chem Inf Model. 2024 Mar 25;64(8):3093–104. doi: 10.1021/acs.jcim.3c02050 (PMC11040721; doi:10.1021/acs.jcim.3c02050)
Supplement: Supplementary file 1 — ci3c02050_si_001.pdf [file ci3c02050_si_001.pdf]

Supporting Information for

Predicting the activity of unidentified chemicals in complementary bioassays from HRMS data to pinpoint potential endocrine disruptors

*Ida Rahu,[1,2], Meelis Kull[1], Anneli Kruve[2,3]**

[1]Institute of Computer Science, University of Tartu, Narva mnt 18, 51009, Tartu, Estonia;
[2]Department of Materials and Environmental Chemistry, Stockholm University, Svante Arrhenius Väg 16, SE-106 91 Stockholm, Sweden;
[3]Department of Environmental Science, Stockholm University, Svante Arrhenius Väg 16, SE-106 91 Stockholm, Sweden
* Corresponding author anneli.kruve@su.se

Contents

Section S1: Tox21 dataset bioassays 2

Section S2: Proportions of the chemicals in the used datasets 3

Section S3: Trained single-output models 5

Section S4: Architectures of the trained multi-output models 6

Section S5: Parameters of the selected models 7

Section S6: Performance of the trained models on the intermediate test set expressed as ROC-AUC and balanced accuracy 9

Section S7: SHAP analysis 11

Section S8: Effect of the Monte Carlo sampling strategy 26

Section S9: Effect of the usage of SIRIUS+CSI:FingerID on the applicability of models 31

References 32

Section S1: Tox21 dataset bioassays

Table S1. Twelve different toxicity assays used in the Tox21 dataset. This table is a modified version of the table from[1].

panel	dataset	toxicity pathway	abbreviation	PubChem AID
nuclear receptor	activators of aryl hydrocarbon receptor	aryl hydrocarbon receptor (AHR) (full receptor) agonism in HepG2 cells	nr.ahr	743122
	activators of androgen receptor	androgen receptor (AR) (full receptor) agonism in MDA-kb-2 cells	nr.ar	743040
	activators of androgen receptor ligand binding domain	AR (partial receptor) agonism in Hek293 cells	nr.ar.lbd	743053
	aromatase inhibitors	inhibition of aromatase in MCF-7 cells	nr.aromatase	743139
	estrogen receptor activators	estrogen receptor (ER) alpha (full receptor) agonism in BG1 cells	nr.er	743079
	activators of estrogen receptor ligand binding domain	ER alpha (partial receptor) agonism in Hek293 cells	nr.er.lbd	743077
	activators of peroxisome proliferator-activated receptor gamma	peroxisome proliferator-activated receptor gamma (PPARg) (partial receptor) agonism in Hek293 cells	nr.ppar.gamma	743140
stress response	activators of antioxidant response element	antioxidant response element (ARE) agonism in HepG2 cells	sr.are	743219
	ATPase family AAA domain-containing protein 5	induced stabilisation of the ATAD5 protein in Hek293 cells	sr.atad5	720516
	activators of heat shock response signaling pathway	heat shock response (HSR) signaling pathway activation in HSE-bla (beta-lactamase reporter gene under the control of heat shock response elements) HeLa cells	sr.hse	743228
	disruptors of mitochondrial membrane potential	mitochondria membrane potential in HepG2 cells	sr.mmp	720637
	activators of p53 signaling pathway	induced stabilization in HCT-116 cells	sr.p53	720552

Section S2: Proportions of the chemicals in the used datasets

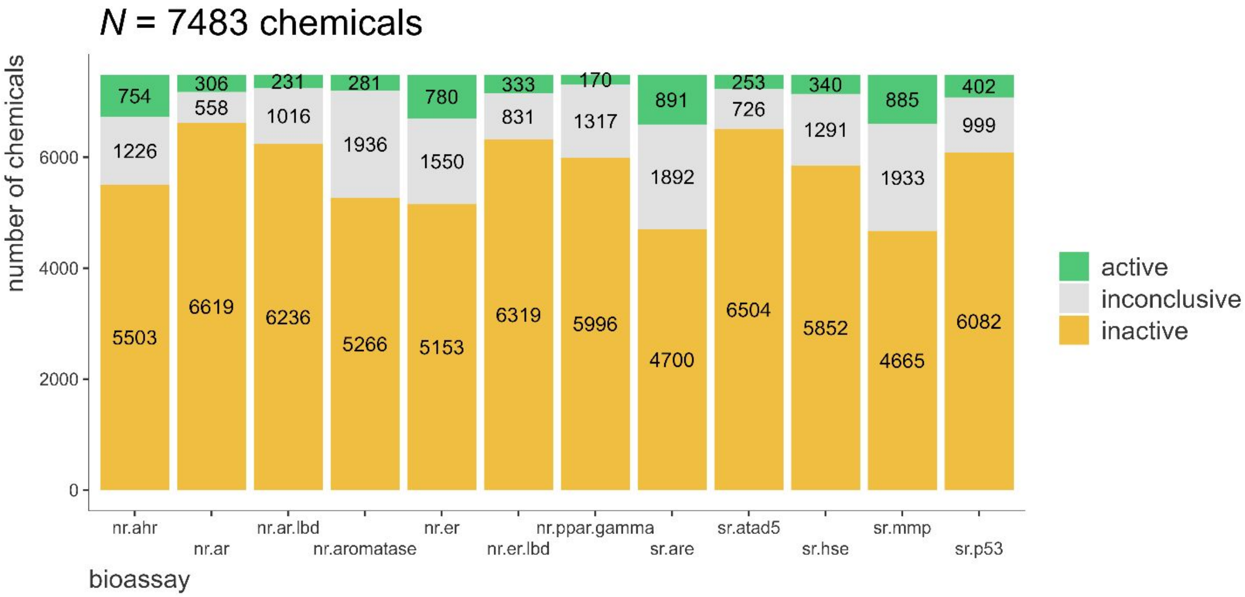

Figure S1. Proportions of the active, inactive, and inconclusive chemicals per bioassay in the original dataset (dataset obtained after deduplication and unsuitable chemicals removal from Tox21 data). This figure is reprinted from[1].

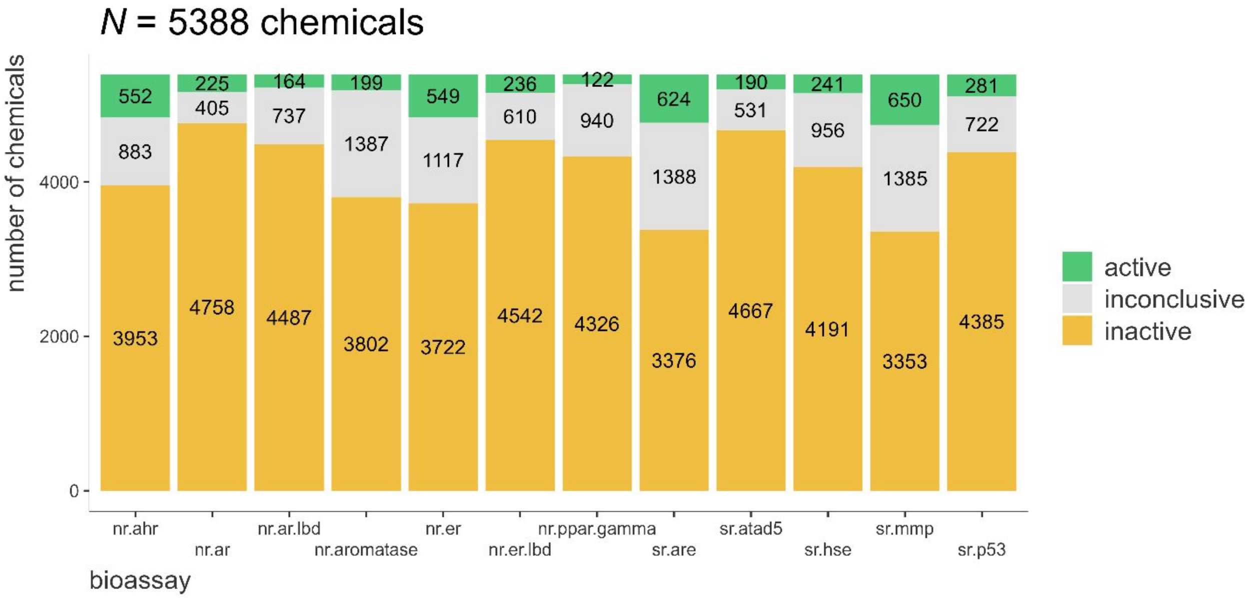

Figure S2. Proportions of the active, inactive, and inconclusive chemicals per bioassay in the training dataset. This figure is reprinted from[1].

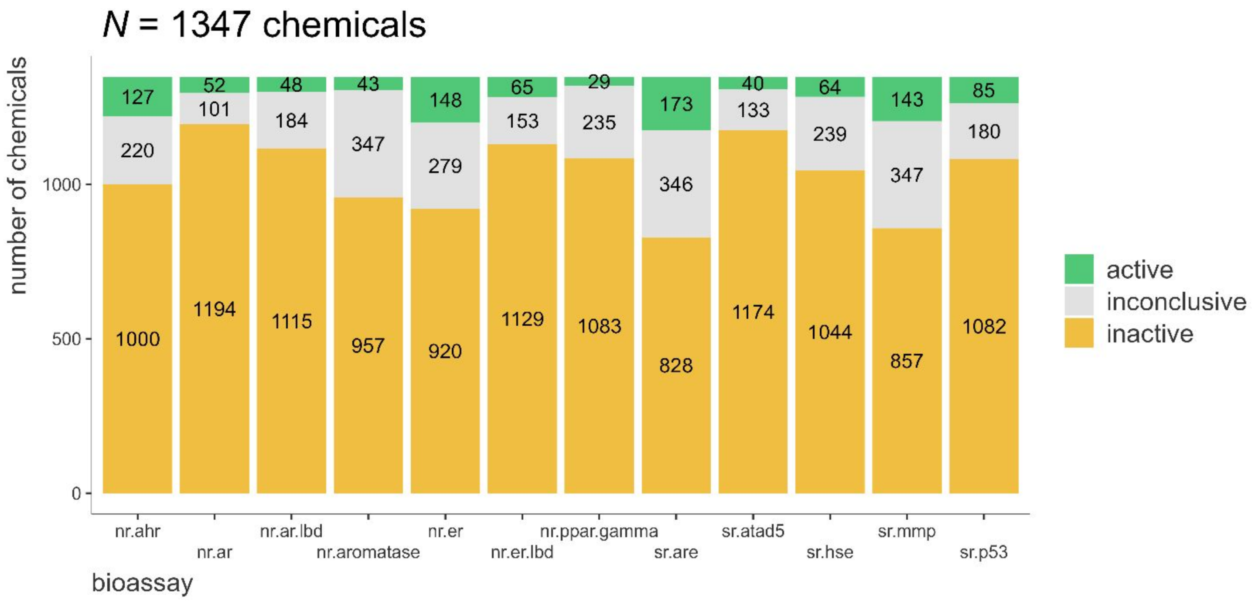

Figure S3. Proportions of the active, inactive, and inconclusive chemicals per bioassay in the intermediate test set. This figure is reprinted from[1].

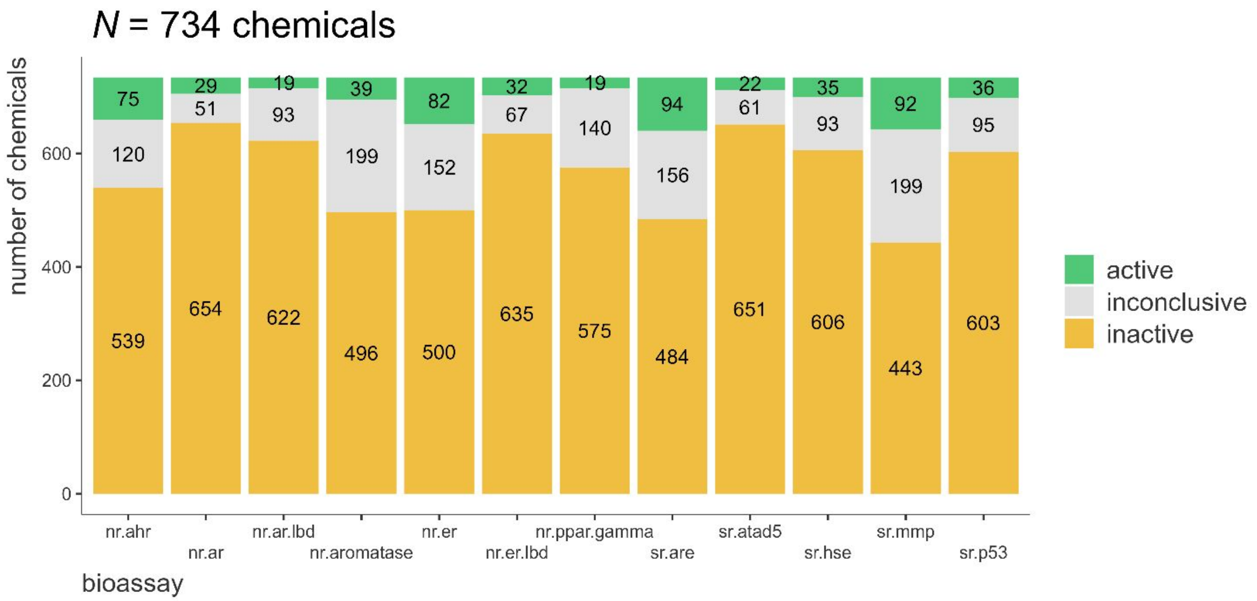

Figure S4. Proportions of the active, inactive, and inconclusive chemicals per bioassay in the real-life test set. This figure is reprinted from[1].

Section S3: Trained single-output models

Table S2. Information about all the trained single-output models[1]. The R package *caret* was utilized for training the models. An automatic grid search approach (with a *tuneLength* parameter of 30) with 10-fold cross-validation was employed to select the optimal set of hyperparameters. This table is reprinted from[1].

model	method name	libraries used	tuning parameters
Bagged AdaBoost	AdaBag	adabag, plyr	mfinal, maxdepth
Bagged CART	treebag	ipred, plyr, e1071	None
Boosted Classification Trees	ada	ada, plyr	iter, maxdepth, nu
Boosted Logistic Regression	LogitBoost	caTools	nIter
C5.0	C5.0	C50, plyr	trials, model, winnow
eXtreme Gradient Boosting	xgbDART	xgboost, plyr	nrounds, max_depth, eta, gamma, subsample, colsample_bytree, rate_drop, skip_drop, min_child_weight
eXtreme Gradient Boosting	xgbTree	xgboost, plyr	nrounds, max_depth, eta, gamma, colsample_bytree, min_child_weight, subsample
k-Nearest Neighbors	kknn	kknn	kmax, distance, kernel
k-Nearest Neighbors	knn		k
Linear Discriminant Analysis	lda	MASS	None
Linear Support Vector Machines with Class Weights	svmLinearWeights	e1071	cost, weight
Naive Bayes	naive_bayes	naivebayes	laplace, usekernel, adjust
Neural Network	nnet	nnet	size, decay
Neural Networks with Feature Extraction	pcaNNet	nnet	size, decay
Random Forest	ranger	e1071, ranger, dplyr	mtry, splitrule, min.node.size
Random Forest	Rborist	Rborist	predFixed, minNode
Random Forest	rf	randomForest	mtry
Regularised Logistic Regression	regLogistic	LiblineaR	cost, loss, epsilon
Stochastic Gradient Boosting	gbm	gbm, plyr	n.trees, interaction.depth, shrinkage, n.minobsinnode

[1] https://topepo.github.io/caret/available-models.html

Section S4: Architectures of the trained multi-output models

Table S3. Proposed architectures of DNNs and considered hyperparameters

considered hyperparameters and architectures	tried values
number of hidden layers	2, 3, 4
number of hidden units per layer	512, 1024, 2048, 4096, 8192
learning rate	0.01, 0.05, 0.1
learning rate reducing factor	0, 0.1
dropout probability	0, 0.3, 0.5
L2 regularisation penalty	0, 10^{-6}, 10^{-5}, 10^{-4}
batch size	32

Section S5: Parameters of the selected models

Table S4. Parameters of the selected single-output models. This table is reprinted from[1].

bioassay	model	parameters	balancing strategy*	cutoff value to remove highly correlated features
nr.ahr	xgbTree	• nrounds = 100<br>• max_depth = 9<br>• eta = 0.3<br>• gamma = 0<br>• colsample_bytree = 0.6<br>• min_child_weight = 1<br>• subsample = 1	down-sampling	0.7
nr.ar.lbd	Rborist	• predFixed = 247<br>• minNode = 2	SMOTE	0.7
nr.ar	gbm	• n.trees = 50<br>• interaction.depth =2<br>• shrinkage = 0.1<br>• n.minobsinnode = 10	None	0.8
nr.aromatase	gbm	• n.trees = 200<br>• interaction.depth = 3<br>• shrinkage = 0.1<br>• n.minobsinnode = 10	up-sampling	0.7
nr.er.lbd	rf	• mtry = 476	None	0.9
nr.er	rf	• mtry = 239	up-sampling	0.9
nr.pppar.gamma	ranger	• mtry =124<br>• splitrule = 'extratrees'<br>• min.node.size =1	SMOTE	0.7
sr.are	xgbDART	• nrounds = 150<br>• max_depth = 9<br>• eta = 0.3<br>• gamma =0<br>• subsample = 1<br>• colsample_bytree = 0.6<br>• rate_drop = 0.01<br>• skip_drop = 0.05<br>• min_child_weight = 1	down-sampling	0.8
sr.atad5	gbm	• n.trees = 150<br>• interaction.depth = 3<br>• shrinkage = 0.1<br>• n.minobsinnode = 10	up-sampling	0.8
sr.hse	gbm	• n.trees = 150<br>• interaction.depth = 3<br>• shrinkage = 0.1<br>• n.minobsinnode = 10	ROSE	0.7
sr.mmp	rf	• mtry = 171	up-sampling	0.8
sr.p53	gbm	• n.trees = 200<br>• interaction.depth = 3<br>• shrinkage = 0.1<br>• n.minobsinnode = 10	down-sampling	0.8

*Sampling methods were employed during training to address the imbalanced data issue. Four different techniques were considered:

- down-sampling
  (function *downSample()* from R package *caret*)
  In the down-sampling approach, a subset of the majority class data points is selected such that the resulting data set has frequencies of the minority and majority classes in close proximity to each other;
- up-sampling
  (function *upSample()* from R package *caret*)
  In up-sampling, the minority class data points are randomly sampled with replacement until their number is identical to the amount of majority class data points;
- synthetic minority over-sampling (SMOTE)
  (function *smote()* from R package *performanceEstimation*)
  The SMOTE method generates new minority class instances by randomly selecting a minority class data point and synthesizing new samples by interpolating the feature values of the selected data point with its *k*-nearest neighbors in the feature space[2];
- random over-sampling (ROSE)
  (function *ROSE()* from R package *ROSE*)
- The ROSE approach generates new synthetic data points by following the smoothed bootstrap technique[3].

The architecture and hyperparameters of the selected multi-output model[1]:

- layers = 3
- units in the first layer = 4096
- units in the second layer = 2048
- units in the third layer = 1024
- learning rate = 0.05
- learning rate reducing factor = 0.1
- dropout probability (for each layer) = 0.5
- L2 regularization penalty = 10^{-6}

Section S6: Performance of the trained models on the intermediate test set expressed as ROC-AUC and balanced accuracy

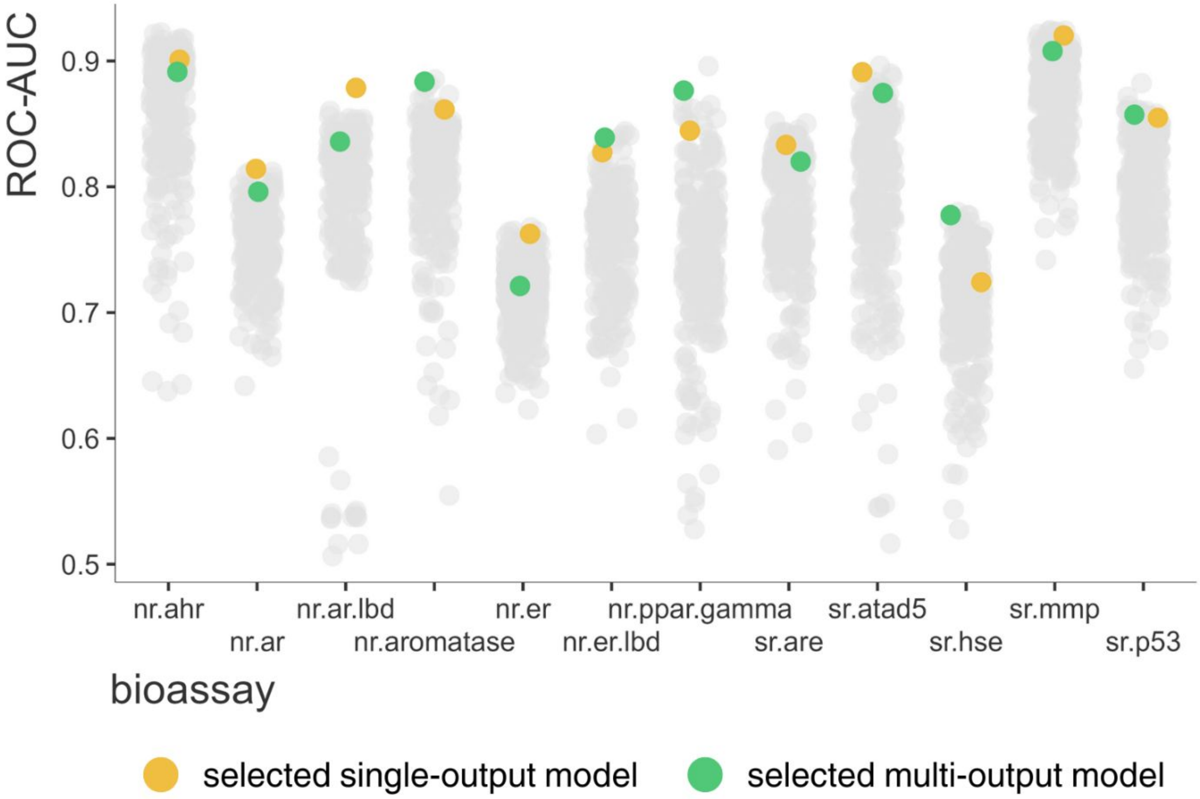

Figure S5. Models' performance on the intermediate test set expressed as ROC-AUC. The data points highlighted in yellow represent the single-output models selected for final evaluation on the real-life test set, while the data points highlighted in green represent the multi-output model chosen for the same purpose. This figure is reprinted from[1].

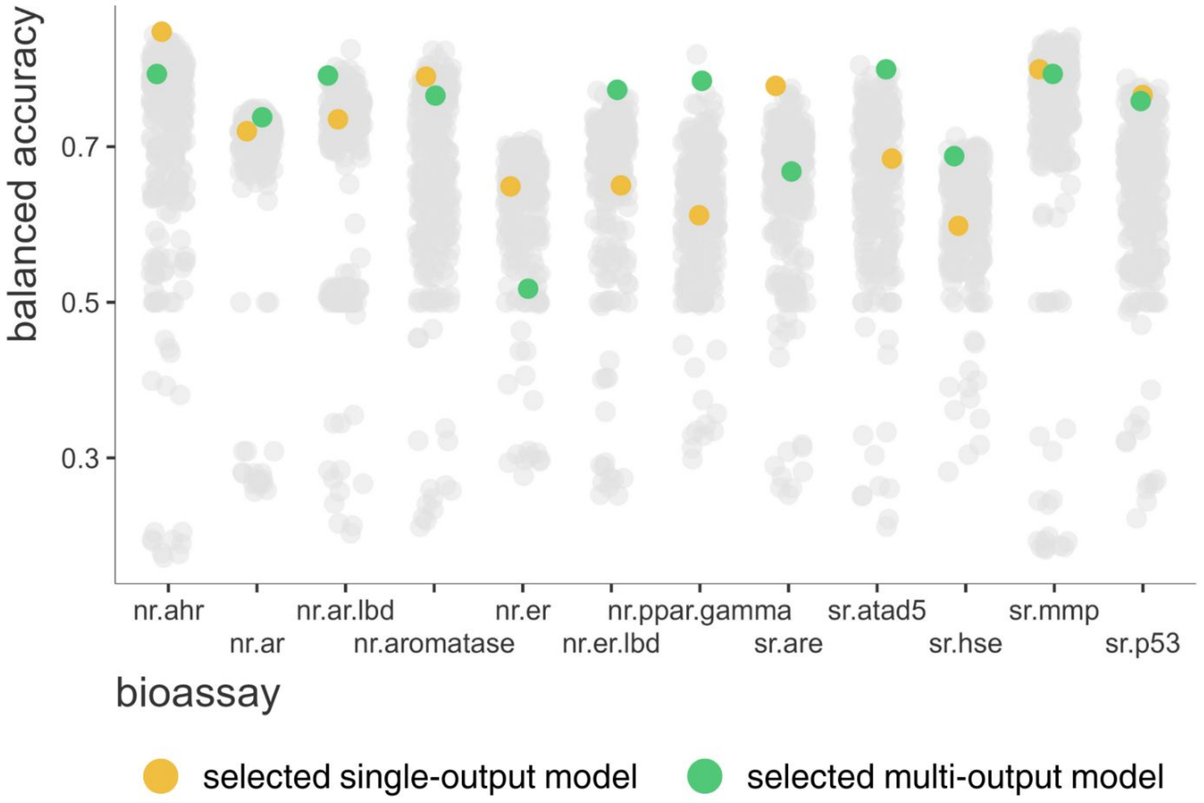

Figure S6. Models' performance on the intermediate test set expressed as balanced accuracy. The data points highlighted in yellow represent the single-output models selected for final evaluation on the real-life test set, while the data points highlighted in green represent the multi-output model chosen for the same purpose. This figure is reprinted from[1].

Section S7: SHAP analysis

Table S5. Top 20 fingerprint features based on the variable importance analysis of three extreme gradient boosting classifiers for the nr.ahr bioassay. These models were trained using distinct sets of fingerprint features acquired by removing highly correlated ones with various cutoff values. Nevertheless, an identical set of chemicals was used for model training, obtained through down-sampling to address data imbalance. The model employing the fingerprint feature set generated with a 0.7 cutoff value underwent final evaluation on the real-life test set. Average SHAP values are provided for the top 20 fingerprint features across all models. A missing value indicates the feature did not rank in the top 20. A "–" symbol denotes the absence of a fingerprint feature in the training data. In the subsequent figures, these fingerprint features are shaded in grey. Features marked in green in the table consistently appeared in all top 20 feature sets. The following figures identify these features with black circles and their respective names.

fingerprint feature	SIRIUS description	mean SHAP value		
		cutoff = 0.7	cutoff = 0.8	cutoff = 0.9
RelIdx_27	C[OH] (28 alcohol)	–	–	0.101
RelIdx_48	[R] (204 Ring)	0.287	0.125	
RelIdx_53	[$([N;!$(N*=[!#6])]);$(N[$([a])]);!$(N~[!#6])] (209 aniline)	0.131		
RelIdx_142	[$([#6X3H0][#6]),$([#6X3H])](=[!#6])[!#6] (Carboxylic_acid_derivative)	–	0.113	0.099
RelIdx_333	[R;$(*(@*)(@*)@*);!$([R2;$(*(@*)(@*)(@*)@*)])]@[R;$(*(@*)(@*)@*);!$([R2;$(*(@*)(@*)(@*)@*)])] (Annelated_rings)	0.241	0.212	0.172
RelIdx_359	[$([CX4;!$([H0]);!$(C[!#6;!$([P,S]=O);!$(N(~O)~O)])][$([CX3]=[O,N,S]),$(C#[N]),$([S,P]=[OX1]),$([NX3]=O),$([NX3+](=O)[O-]);!$(*[S,O,N;H1,H2]);!$([*+0][S,O;X1-])]),$([CX4;!$([H0])]1[CX3]=[CX3][CX3]=[CX3]1)] (CH-acidic)	0.116	0.086	
RelIdx_467	[!#6;!#1]~*(~[!#6;!#1])~[!#6;!#1]	0.116	0.118	
RelIdx_473	*~*(~*)(~*)~*		0.099	0.111
RelIdx_475	[CH3]~[CH2]~*		0.092	
RelIdx_486	unidentified	–	0.145	0.107
RelIdx_490	[$(*~[CH2]~*~*~[CH2]~*),$([R]1@[CH2]@[R]@[R]@[CH2;R]1),$(*~[CH2]~[R]1@[R]@[CH2;R]1)]	–	0.177	
RelIdx_494	*@*!@[#7]	0.149		
RelIdx_500	[O;!H0]	0.146	0.092	
RelIdx_505	*!:*:*!:*	0.118	0.176	0.119
RelIdx_513	[#8]~[#6](~[#6])~[#6]	0.249	0.323	0.206
RelIdx_517	[#7]~*(~*)~*	–	–	0.133
RelIdx_707	>= 1 saturated or aromatic carbon-only ring size 6	–	0.227	
RelIdx_714	>= 2 saturated or aromatic carbon-only ring size 6	0.120		
RelIdx_785	>= 2 aromatic rings	–	–	0.213
RelIdx_914	[#6](:c)(:c)(:n) (C(:C)(:C)(:N))	0.165		0.091
RelIdx_921	[#7&!H0](~[#6]) (N(~C)(~H))	–	–	0.142
RelIdx_960	[#6](-,:[#6])(-,:[#6])(=,:[#8]) (C(-C)(-C)(=O))	0.127		
RelIdx_1030	[#7]-,:[#6]=,:[#6&!H0] (N-C=C-[#1])	–	0.346	0.156
RelIdx_1099	[#6&!H0]-,:[#8&!H0] ([#1]-C-O-[#1])	0.147		
RelIdx_1127	[#6H,#6H2,#6H3]-,:[#6]=,:[#6H,#6H2,#6H3] ([#1]-C-C=C-[#1])	0.134		
RelIdx_1128	[#7]-,:[#6]:[#6]:[#6]-,:[#6] (N-C:C:C-C)	–	–	0.318
RelIdx_1165	[#8]-,:[#6]-,:[#6]-,:[#6]-,:[#6] (O-C-C-C-C)	–	0.154	
RelIdx_1171	[#6&!H0]-,:[#6]-,:[#7&!H0] ([#1]-C-C-N-[#1])	–	0.152	

fingerprint feature	SIRIUS description	mean SHAP value		
		cutoff = 0.7	cutoff = 0.8	cutoff = 0.9
RelIdx_1200	[#8]=,:[#6]-,:[#6]=,:[#6&!H0] (O=C-C=C-[#1])	0.127		
RelIdx_1965	[!#1][CH2]C(=O)O[!#1]			0.089
RelIdx_3053	[!#1]c1[cH][cH]c([!#1])c([!#1])[cH]1	0.114		
RelIdx_4632	CC		0.096	
RelIdx_5048	CCC	0.199		0.090
RelIdx_5109	CCCC=O	–	–	0.088
RelIdx_5114	CCCCC	0.192	0.118	
RelIdx_5120	CCCCCC	–	–	0.086
RelIdx_5334	CNC	–	–	0.106
RelIdx_5351	CNc1ccccc1	0.129	–	
RelIdx_8421	c[CH0]	0.177	0.097	0.085
RelIdx_8469	c(:c:c:c:c:1):c:1	–	–	0.393
RelIdx_8524	c(:c:c:c:c:1):c:1~[#7]	–	0.232	

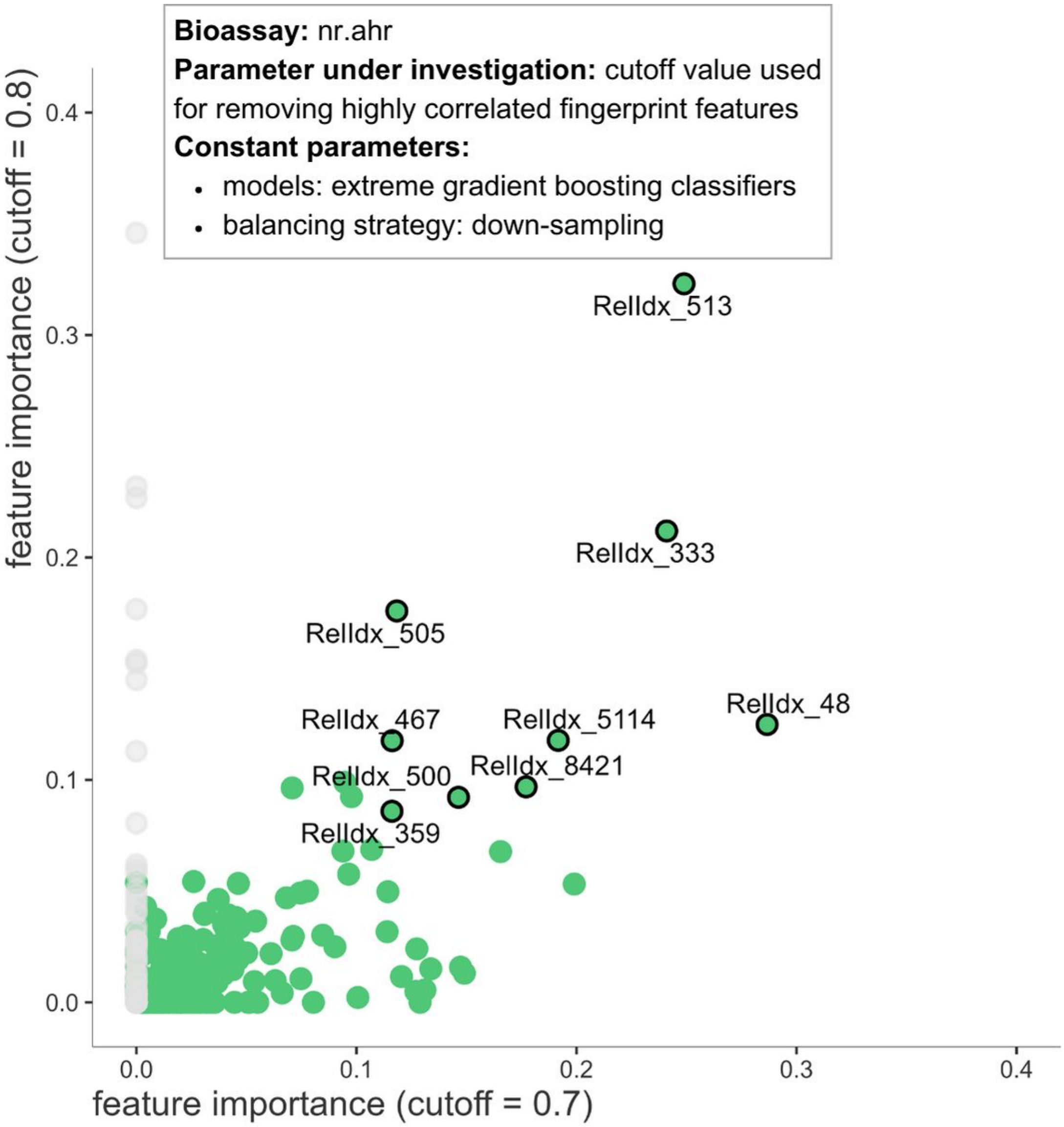

Figure S7. Comparative analysis of results of variable importance analysis of two extreme gradient boosting classifiers trained for predicting the nr.ahr bioassay endpoint. These models were trained using different sets of fingerprint features obtained by removing highly correlated ones with varying cutoff values. However, the model training employed an identical set of chemicals obtained through down-sampling to address data imbalance. In the plot, the *x*-axis represents the average SHAP values of the model trained with a feature set obtained using a 0.7 cutoff value, while the *y*-axis represents the average SHAP values of the model trained with a feature set obtained using a 0.8 cutoff value. The fingerprint features highlighted in gray correspond to those that were absent from the fingerprint feature set used in training the model with the lower cutoff value. Features marked with black circles and their respective names are those that ranked among the top 20 most important features for both models (see **Table S5**).

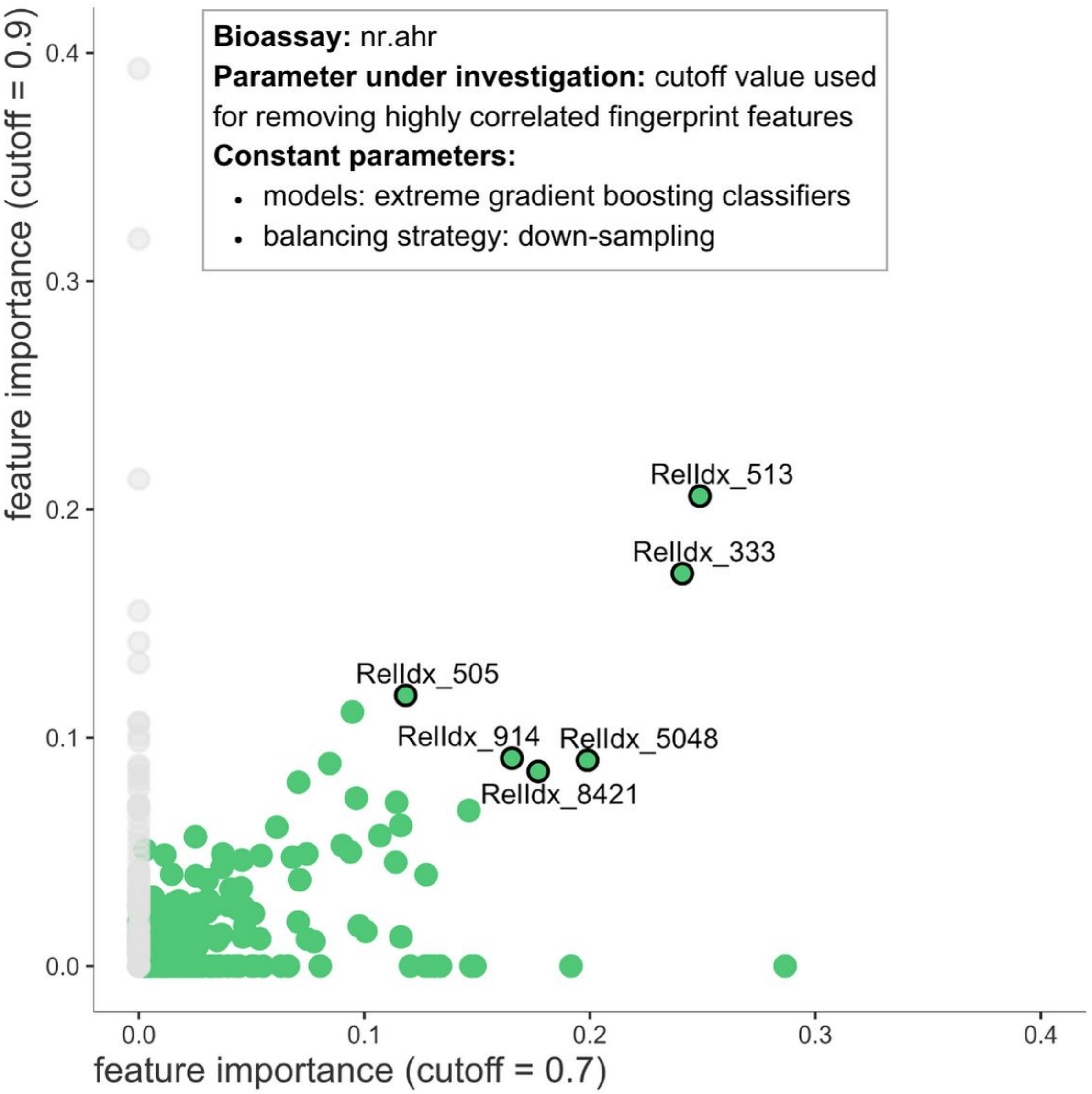

Figure S8. Comparative analysis of results of variable importance analysis of two extreme gradient boosting classifiers trained for predicting the nr.ahr bioassay endpoint. These models were trained using different sets of fingerprint features obtained by removing highly correlated ones with varying cutoff values. However, the model training employed an identical set of chemicals obtained through down-sampling to address data imbalance. In the plot, the *x*-axis represents the average SHAP values of the model trained with a feature set obtained using a 0.7 cutoff value, while the *y*-axis represents the average SHAP values of the model trained with a feature set obtained using a 0.9 cutoff value. The fingerprint features highlighted in gray correspond to those that were absent from the fingerprint feature set used in training the model with the lower cutoff value. Features marked with black circles and their respective names are those that ranked among the top 20 most important features for both models (see **Table S5**).

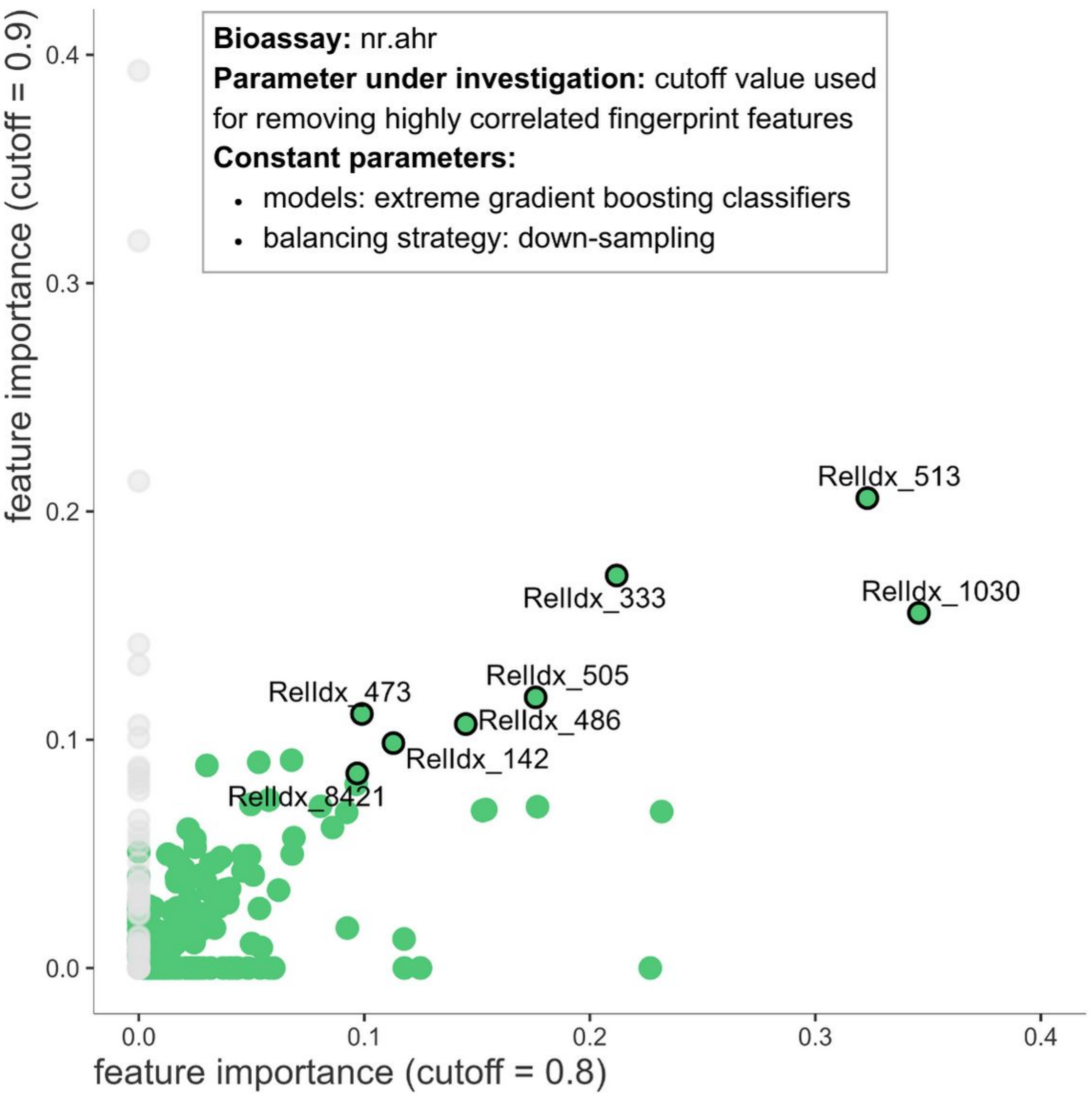

Figure S9. Comparative analysis of results of variable importance analysis of two extreme gradient boosting classifiers trained for predicting the nr.ahr bioassay endpoint. These models were trained using different sets of fingerprint features obtained by removing highly correlated ones with varying cutoff values. However, the model training employed an identical set of chemicals obtained through down-sampling to address data imbalance. In the plot, the *x*-axis represents the average SHAP values of the model trained with a feature set obtained using a 0.8 cutoff value, while the *y*-axis represents the average SHAP values of the model trained with a feature set obtained using a 0.9 cutoff value. The fingerprint features highlighted in gray correspond to those that were absent from the fingerprint feature set used in training the model with the lower cutoff value. Features marked with black circles and their respective names are those that ranked among the top 20 most important features for both models (see **Table S5**).

Table S6. Top 20 fingerprint features based on the variable importance analysis of extreme gradient boosting and random forest classifiers for the nr.ahr bioassay. These models are trained on identical sets of fingerprint features (obtained using a cutoff value of 0.7 for removing highly correlated features) and chemicals (obtained employing a down-sampling technique to address the data imbalance). The model that utilizes the extreme gradient boosting algorithm underwent final evaluation on the real-life test set. Average SHAP values are provided for the top 20 fingerprint features across both models. A missing value indicates the feature did not rank in the top 20. Fingerprint features marked in green in the table consistently appeared in both top 20 feature sets. The following figures identify these features with black circles and their respective names.

fingerprint feature	SIRIUS description	mean SHAP value	
		boosting	RF
RelIdx_48	[R] (204 Ring)	0.287	0.044
RelIdx_53	[$([N;!$(N*=[!#6])]);$(N[$([a])]);!$(N~[!#6])] (209 aniline)	0.131	0.008
RelIdx_56	[CX4H2]([#6])[#6] (Secondary_carbon)		0.006
RelIdx_333	[R;$(*(@*)(@*)@*);!$([R2;$(*(@*)(@*)(@*)@*)])]@[R;$(*(@*)(@*)@*);!$([R2;$(*(@*)(@*)(@*)@*)])] (Annelated_rings)	0.241	0.027
RelIdx_359	[$([CX4;!$([H0]);!$(C[!#6;!$([P,S]=O);!$(N(~O)~O)])][$([CX3]=[O,N,S]),$(C#[N]),$([S,P]=[OX1]),$([NX3]=O),$([NX3+](=O)[O-]);!$(*[S,O,N;H1,H2]);!$([*+0][S,O;X1-])]),$([CX4;!$([H0])]1[CX3]=[CX3][CX3]=[CX3]1)] (CH-acidic)	0.116	
RelIdx_413	[#7]~[#7]		0.007
RelIdx_467	[!#6;!#1]~*(~[!#6;!#1])~[!#6;!#1]	0.116	0.007
RelIdx_469	[CH3]~*~*~*~[CH2]~*		0.006
RelIdx_494	*@*!@[#7]	0.149	0.025
RelIdx_500	[O;!H0]	0.146	
RelIdx_505	*!:*:*!:*	0.118	0.018
RelIdx_513	[#8]~[#6](~[#6])~[#6]	0.249	
RelIdx_521	[C;H3,H4]		0.010
RelIdx_714	>= 2 saturated or aromatic carbon-only ring size 6	0.120	
RelIdx_870	[#6](~[#6])(~[Cl]) (C(~C)(~Cl))		0.008
RelIdx_914	[#6](:c)(:c)(:n) (C(:C)(:C)(:N))	0.165	
RelIdx_960	[#6](-,:[#6])(-,:[#6])(=,:[#8]) (C(-C)(-C)(=O))	0.127	
RelIdx_1099	[#6&!H0]-,:[#8&!H0] ([#1]-C-O-[#1])	0.147	
RelIdx_1104	[#7]=,:[#6]-,:[#6]:[#6&!H0] (N=C-C:C-[#1])		0.008
RelIdx_1127	[#6H,#6H2,#6H3]-,:[#6]=,:[#6H,#6H2,#6H3] ([#1]-C-C=C-[#1])	0.134	0.051
RelIdx_1200	[#8]=,:[#6]-,:[#6]=,:[#6&!H0] (O=C-C=C-[#1])	0.127	
RelIdx_1207	[#6]-,:[#6]-,:[#6]-,:[#6]-,:[#6]-,:[#6] (C-C-C-C-C-C)		0.009
RelIdx_3053	[!#1]c1[cH][cH]c([!#1])c([!#1])[cH]1	0.114	
RelIdx_4632	CC		0.008
RelIdx_5048	CCC	0.199	0.020
RelIdx_5114	CCCCC	0.192	0.029
RelIdx_5130	CCCCCCCC		0.011
RelIdx_5351	CNc1ccccc1	0.129	0.008
RelIdx_7270	ECFP6:87183595		0.008
RelIdx_8421	c[CH0]	0.177	

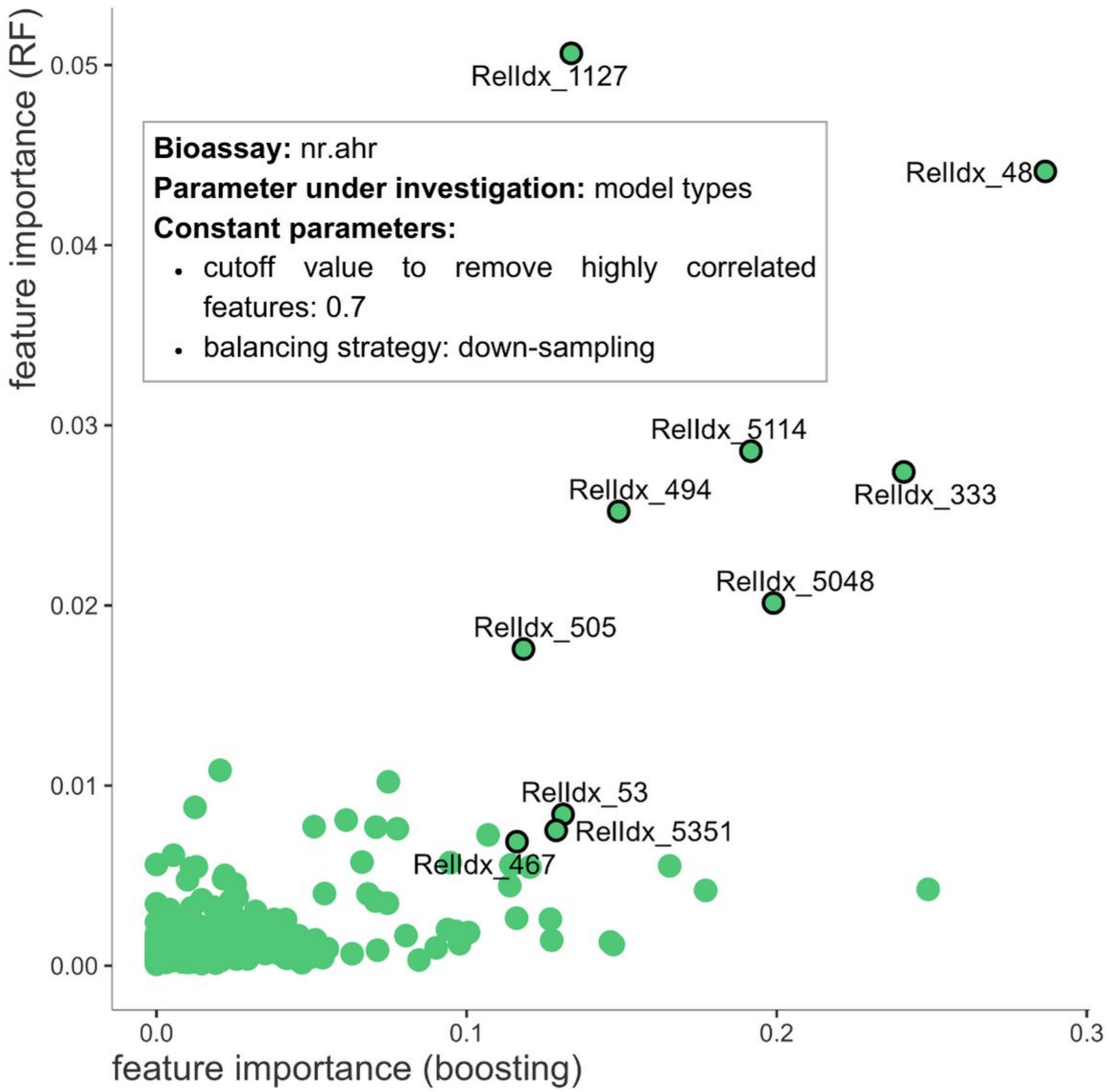

Figure S10. Comparative analysis of results of variable importance analysis of extreme gradient boosting and random forest classifiers trained for predicting the nr.ahr bioassay endpoint. These models are trained on identical sets of fingerprint features (obtained using a cutoff value of 0.7 for removing highly correlated features) and chemicals (obtained employing a down-sampling technique to address the data imbalance). In the plot, the *x*-axis represents the average SHAP values of the extreme gradient boosting classifier, while the *y*-axis represents the average SHAP values of the random forest classifier. Fingerprint features marked with black circles and their respective names correspond to those that ranked among the top 20 most important features for both models (see **Table S6**).

Table S7. Top 20 fingerprint features based on the variable importance analysis of the three random forest classifiers for the nr.er bioassay. These models were trained using distinct sets of fingerprint features acquired by removing highly correlated ones with various cutoff values. Nevertheless, an identical set of chemicals was used for model training, obtained through up-sampling to address data imbalance. The model employing the fingerprint feature set generated with a 0.9 cutoff value underwent final evaluation on the real-life test set. Average SHAP values are provided for the top 20 fingerprint features across all models. A missing value indicates the feature did not rank in the top 20. A "–" symbol denotes the absence of a fingerprint feature in the training data. In the subsequent figures, these fingerprint features are shaded in grey. Features marked in green in the table consistently appeared in all top 20 feature sets. The following figures identify these features with black circles and their respective names.

fingerprint feature	SIRIUS description	mean SHAP value		
		cutoff = 0.7	cutoff = 0.8	cutoff = 0.9
RelIdx_33	c[OH] (34 phenol)	0.005		
RelIdx_48	[R] (204 Ring)	0.005		
RelIdx_49	[$([$([C;$(C=[$([O;D1;$(O=C)])])]);$(C[$([O;$([H1&-0,H0&-1])])]);$(C[#6,#1])])] (205 carboxylic acid)		0.004	0.003
RelIdx_55	[CX4H3][#6] (Primary_carbon)	–		0.003
RelIdx_354	[$([#7X2,OX1,SX1]=*[!H0;!$([a;!n])]) ,$([#7X3,OX2,SX2;!H0]*=*), $([#7X3,OX2,SX2;!H0]*:n)] (1,3-Tautomerizable)		0.004	0.003
RelIdx_461	*~[CH2]~[#7]	0.010	0.004	0.003
RelIdx_465	[!#6;!#1;!H0]~*~[CH2]~*	0.006	0.004	
RelIdx_472	[#7]~*~[CH2]~*	0.004		
RelIdx_494	*@*!@[#7]	0.004		
RelIdx_497	[#8]=* >= 2	0.005	0.004	0.003
RelIdx_499	[!#6;!#1]~[CH2]~* >= 2	0.005		
RelIdx_505	*!:*:*!:*			0.003
RelIdx_506	*1~*~*~*~*~*~1 >= 2	–	0.016	0.019
RelIdx_510	[C;H3,H4] >= 2	0.004		
RelIdx_513	[#8]~[#6](~[#6])~[#6]	0.014	0.007	0.006
RelIdx_516	*!@[CH2]!@*		0.003	
RelIdx_707	>= 1 saturated or aromatic carbon-only ring size 6	–	0.014	0.012
RelIdx_714	>= 2 saturated or aromatic carbon-only ring size 6	0.045	0.035	0.037
RelIdx_720	>= 3 any ring size 6	0.005	0.005	0.004
RelIdx_873	[#6&!H0](~[#6])(~[#7]) (C(~C)(~H)(~N))	–	0.014	0.013
RelIdx_1044	[#6H,#6H2,#6H3]=,:[#6H,#6H2,#6H3] ([#1]-C=C-[#1])	–	0.009	0.007
RelIdx_1127	[#6H,#6H2,#6H3]-,:[#6]=,:[#6H,#6H2,#6H3] ([#1]-C-C=C-[#1])	0.017	0.007	0.005
RelIdx_1184	[#6]-,:[#6]-,:[#7]-,:[#6]-,:[#6] (C-C-N-C-C)	–	0.003	
RelIdx_1207	[#6]-,:[#6]-,:[#6]-,:[#6]-,:[#6]-,:[#6] (C-C-C-C-C-C)	0.004		
RelIdx_1388	[#6][#6]1[#6]([#6])[#6][#6][#6]1 (CC1C(C)CCC1)	0.004		
RelIdx_3050	[!#1]c1[cH][cH]c([!#1])[cH][cH]1	0.007	0.004	0.004
RelIdx_5133	CCCCCCCC=O		0.004	0.003
RelIdx_5157	CCl	0.004	0.003	0.003

fingerprint feature	SIRIUS description	mean SHAP value		
		cutoff = 0.7	cutoff = 0.8	cutoff = 0.9
RelIdx_5158	CCN	–	0.004	0.003
RelIdx_7834	ECFP6:1323701668	0.012	0.007	0.004
RelIdx_8148	ECFP6:2078126852	0.004		
RelIdx_8427	[CH1][CH2][CH2][CH0]	0.004		
RelIdx_8469	c(:c:c:c:c:1):c:1	–	–	0.007

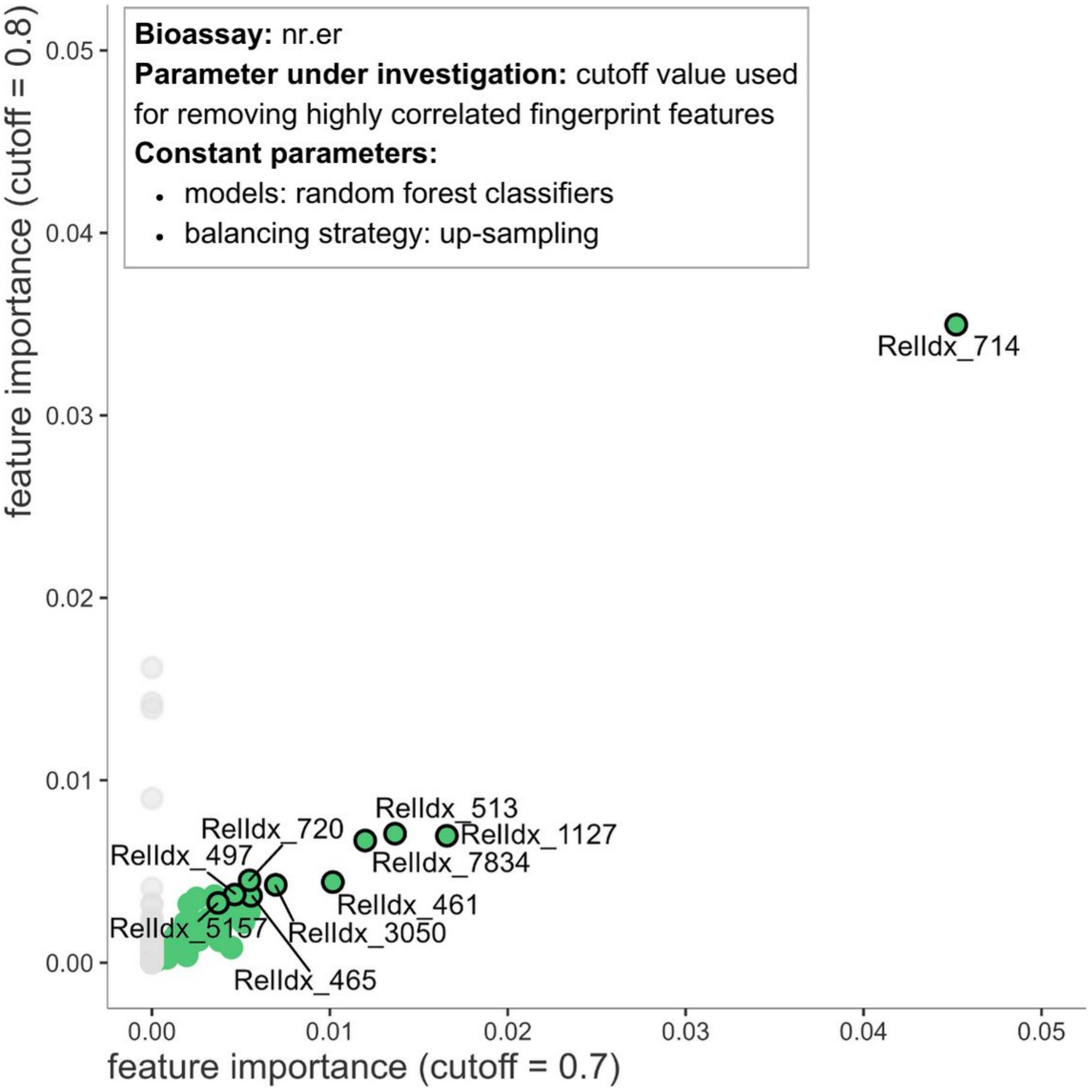

Figure S11. Comparative analysis of results of variable importance analysis of two random forest classifiers trained for predicting the nr.er bioassay endpoint. These models were trained using different sets of fingerprint features obtained by removing highly correlated ones with varying cutoff values. However, the model training employed an identical set of chemicals obtained through up-sampling to address data imbalance. In the plot, the *x*-axis represents the average SHAP values of the model trained with a feature set obtained using a 0.7 cutoff value, while the *y*-axis represents the average SHAP values of the model trained with a feature set obtained using a 0.8 cutoff value. The fingerprint features highlighted in gray correspond to those that were absent from the fingerprint feature set used in training the model with the lower cutoff value. Features marked with black circles and their respective names are those that ranked among the top 20 most important features for both models (see **Table S7**).

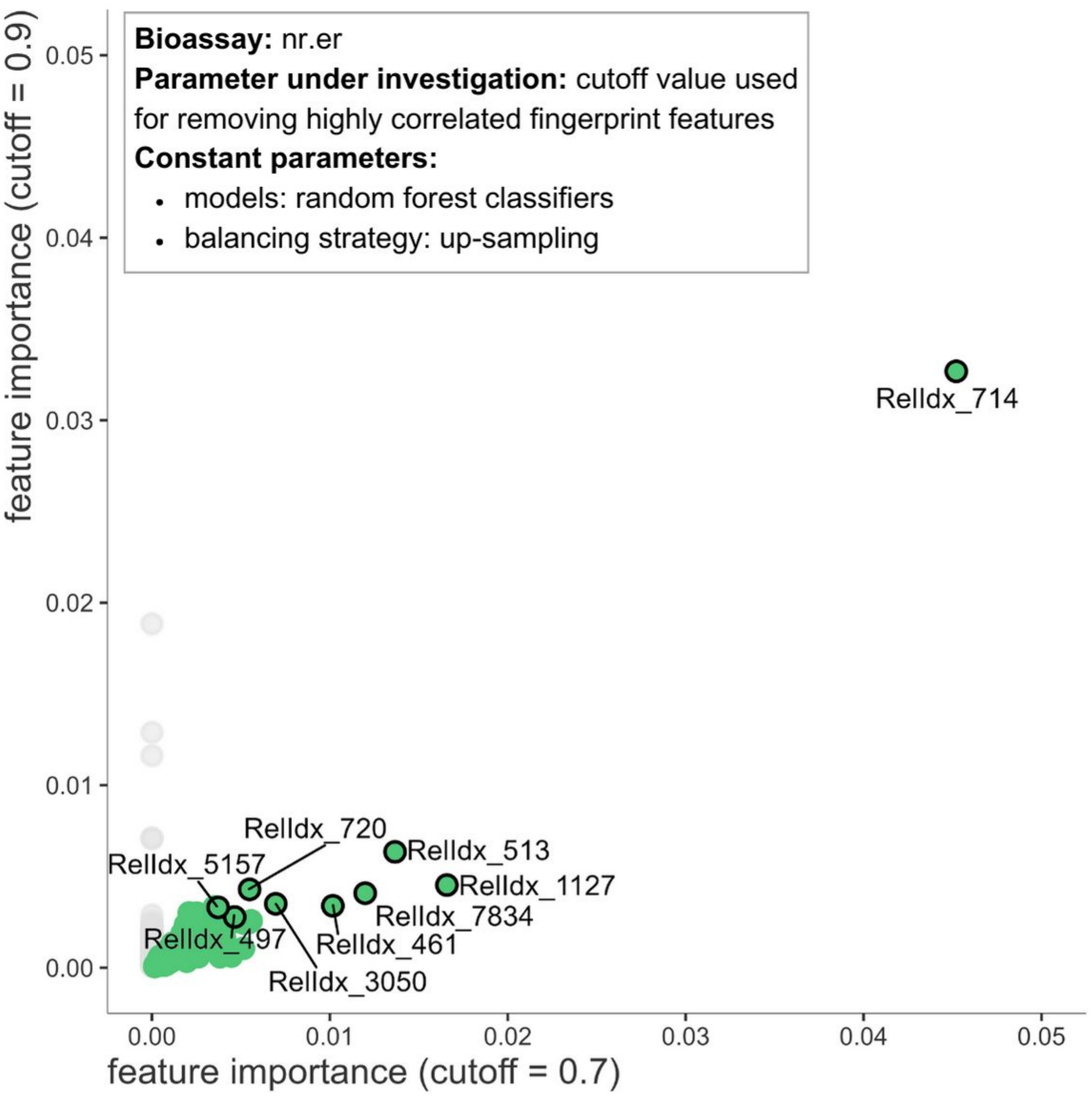

Figure S12. Comparative analysis of results of variable importance analysis of two random forest classifiers trained for predicting the nr.er bioassay endpoint. These models were trained using different sets of fingerprint features obtained by removing highly correlated ones with varying cutoff values. However, the model training employed an identical set of chemicals obtained through up-sampling to address data imbalance. In the plot, the *x*-axis represents the average SHAP values of the model trained with a feature set obtained using a 0.7 cutoff value, while the *y*-axis represents the average SHAP values of the model trained with a feature set obtained using a 0.9 cutoff value. The fingerprint features highlighted in gray correspond to those that were absent from the fingerprint feature set used in training the model with the lower cutoff value. Features marked with black circles and their respective names are those that ranked among the top 20 most important features for both models (see **Table S7**).

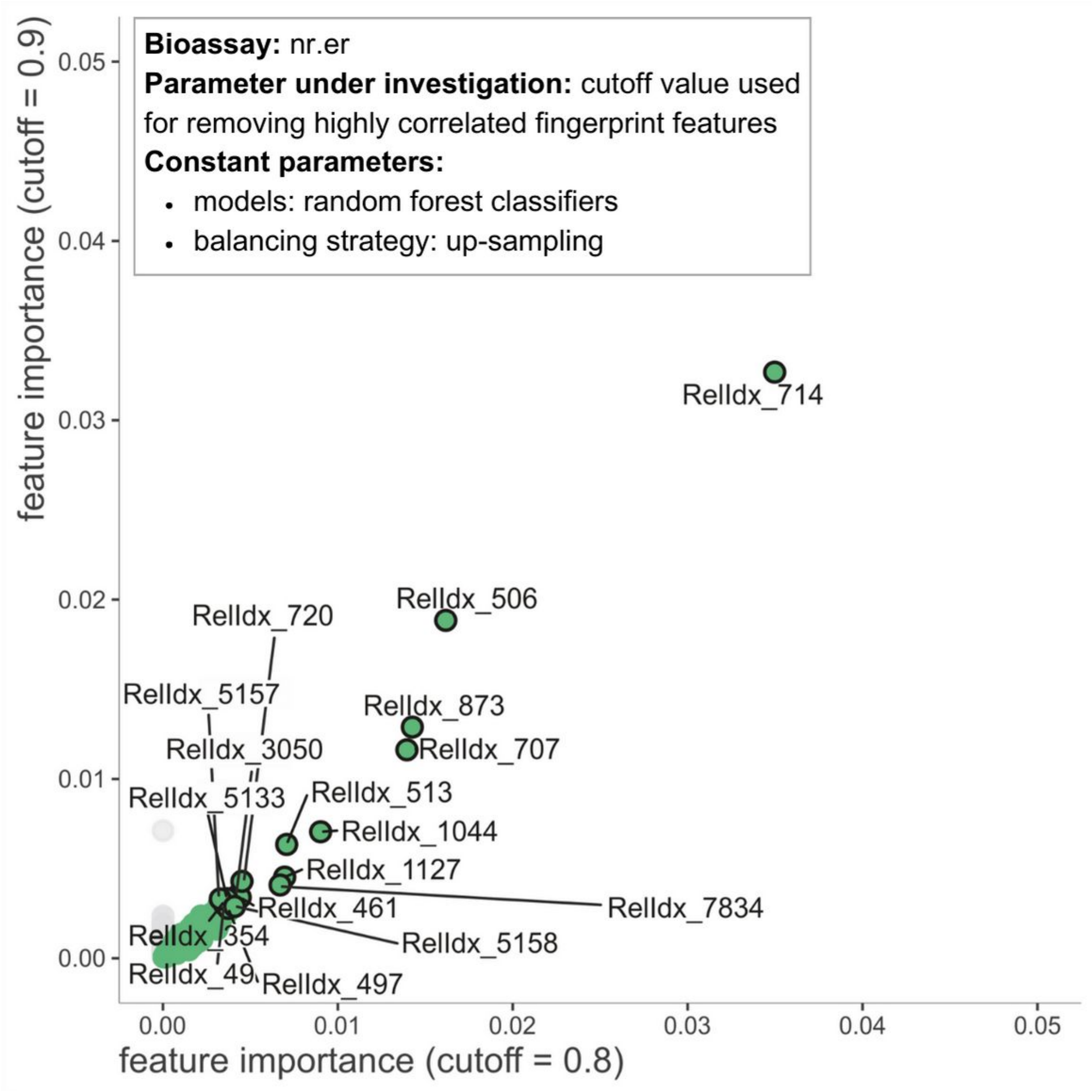

Figure S13. Comparative analysis of results of variable importance analysis of two random forest classifiers trained for predicting the nr.er bioassay endpoint. These models were trained using different sets of fingerprint features obtained by removing highly correlated ones with varying cutoff values. However, the model training employed an identical set of chemicals obtained through up-sampling to address data imbalance. In the plot, the *x*-axis represents the average SHAP values of the model trained with a feature set obtained using a 0.8 cutoff value, while the *y*-axis represents the average SHAP values of the model trained with a feature set obtained using a 0.9 cutoff value. The fingerprint features highlighted in gray correspond to those that were absent from the fingerprint feature set used in training the model with the lower cutoff value. Features marked with black circles and their respective names are those that ranked among the top 20 most important features for both models (see **Table S7**).

Table S8. Top 20 fingerprint features based on the variable importance analysis of random forest and extreme gradient boosting classifiers for the nr.er bioassay. These models are trained on identical sets of fingerprint features (obtained using a cutoff value of 0.9 for removing highly correlated features) and chemicals (obtained employing an up-sampling technique to address the data imbalance). The model that utilizes the random forest algorithm underwent final evaluation on the real-life test set. Average SHAP values are provided for the top 20 fingerprint features across both models. A missing value indicates the feature did not rank in the top 20. Fingerprint features marked in green in the table consistently appeared in both top 20 feature sets. The following figures identify these features with black circles and their respective names.

fingerprint feature	SIRIUS description	mean SHAP value	
		RF	boosting
RelIdx_49	[$([$([C;$(C=[$([O;D1;$(O=C)])])]);$(C[$([O;$([H1&-0,H0&-1])])]);$(C[#6,#1])])] (205 carboxylic acid)	0.003	
RelIdx_55	[CX4H3][#6] (Primary_carbon)	0.003	
RelIdx_354	[$([#7X2,OX1,SX1]=*[!H0;!$([a;!n])]),$([#7X3,OX2,SX2;!H0]*=*),$([#7X3,OX2,SX2;!H0]*:n)] (1,3-Tautomerizable)	0.003	
RelIdx_361	[$([*@](~*)(~*)(*)*),$([*@H](*)(*)*),$([*@](~*)(*)*),$([*@H](~*)~*)] (Chiral_center_specified)		0.086
RelIdx_441	[#7]~*~*~*~[#7]		0.091
RelIdx_461	*~[CH2]~[#7]	0.003	
RelIdx_465	[!#6;!#1;!H0]~*~[CH2]~*		0.097
RelIdx_490	[$(*~[CH2]~*~*~[CH2]~*),$([R]1@[CH2]@[R]@[R]@[CH2;R]1),$(*~[CH2]~[R]1@[R]@[CH2;R]1)]		0.084
RelIdx_494	*@*!@[#7]		0.099
RelIdx_497	[#8]=* >= 2	0.003	
RelIdx_505	*!:*:*!:*	0.003	
RelIdx_506	*1~*~*~*~*~*~1 >= 2	0.019	0.170
RelIdx_510	[C;H3,H4] >= 2		0.109
RelIdx_513	[#8]~[#6](~[#6])~[#6]	0.006	0.250
RelIdx_707	>= 1 saturated or aromatic carbon-only ring size 6	0.012	0.108
RelIdx_712	>= 1 unsaturated non-aromatic heteroatom-containing ring size 6		0.083
RelIdx_714	>= 2 saturated or aromatic carbon-only ring size 6	0.033	0.282
RelIdx_720	>= 3 any ring size 6	0.004	0.128
RelIdx_873	[#6&!H0](~[#6])(~[#7]) (C(~C)(~H)(~N))	0.013	0.083
RelIdx_958	[#6](-,:[#6])(-,:[#6])(=,:[#6]) (C(-C)(-C)(=C))		0.109
RelIdx_1044	[#6H,#6H2,#6H3]=,:[#6H,#6H2,#6H3] ([#1]-C=C-[#1])	0.007	0.171
RelIdx_1127	[#6H,#6H2,#6H3]-,:[#6]=,:[#6H,#6H2,#6H3] ([#1]-C-C=C-[#1])	0.005	
RelIdx_1240	[#6]-,:[#6](-,:[#6])-,:[#6](-,:[#6])-,:[#6] (C-C(C)-C(C)-C)		0.109
RelIdx_1347	[#6][#6]1[#6]([#8])[#6][#6][#6][#6]1 (CC1C(O)CCCC1)		0.136
RelIdx_1409	[!#1][CH]([!#1])[!#1] ([!#1][CH]([!#1])[!#1])		0.084
RelIdx_3050	[!#1]c1[cH][cH]c([!#1])[cH][cH]1	0.004	

fingerprint feature	SIRIUS description	mean SHAP value	
		RF	boosting
RelIdx_5133	CCCCCCCC=O	0.003	
RelIdx_5152	CCCO		0.099
RelIdx_5157	CCl	0.003	
RelIdx_5158	CCN	0.003	
RelIdx_7834	ECFP6:1323701668	0.004	0.118
RelIdx_8469	c(:c:c:c:c:1):c:1	0.007	

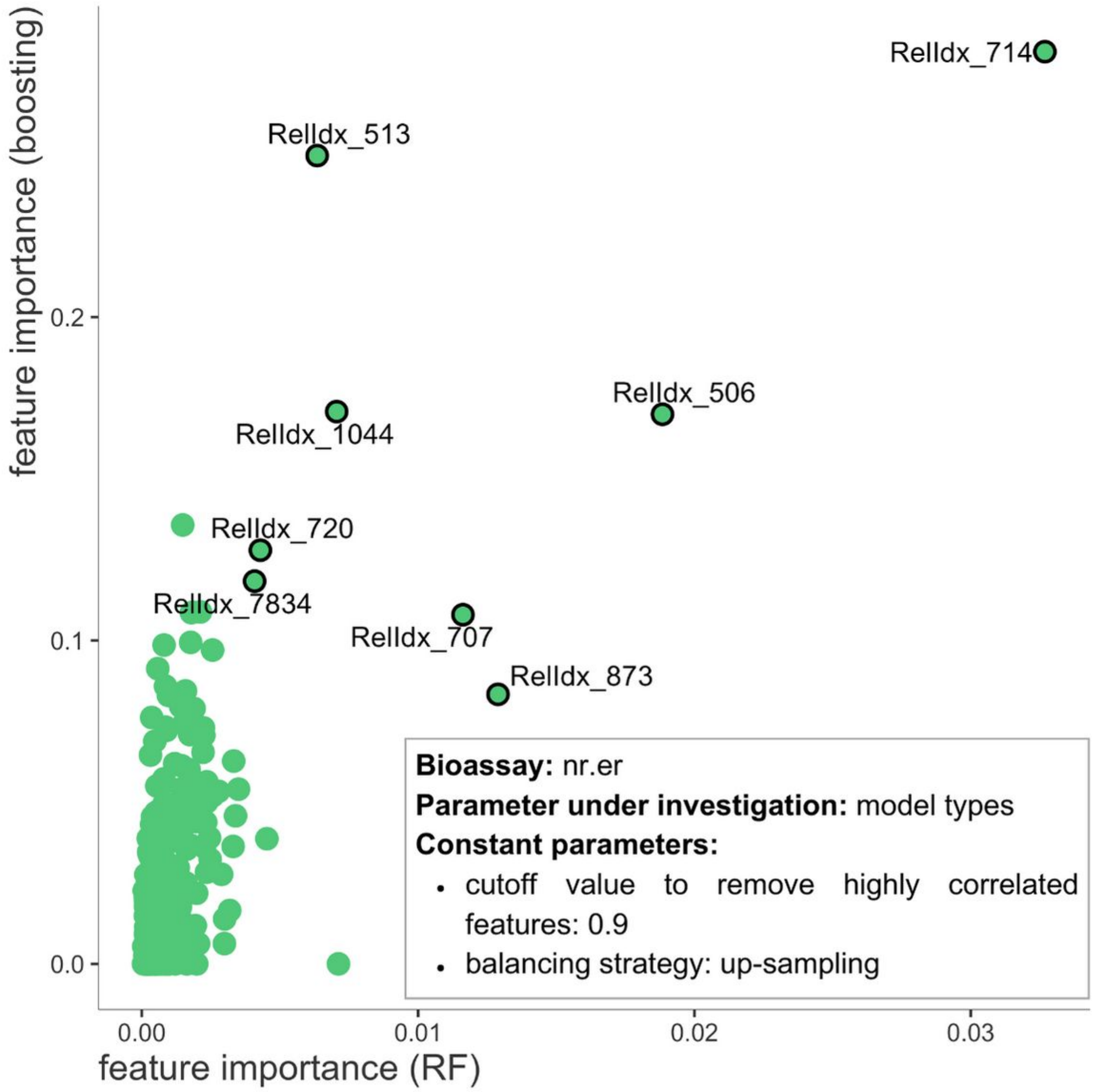

Figure S14. Comparative analysis of results of variable importance analysis of random forest and extreme gradient boosting classifiers trained for predicting the nr.er bioassay endpoint. These models are trained on identical sets of fingerprint features (obtained using a cutoff value of 0.9 for removing highly correlated features) and chemicals (obtained employing an up-sampling technique to address the data imbalance). In the plot, the *x*-axis represents the average SHAP values of the random forest classifier, while the *y*-axis represents the average SHAP values of the extreme gradient boosting classifier. Fingerprint features marked with black circles and their respective names correspond to those that ranked among the top 20 most important features for both models (see **Table S8**).

Section S8: Effect of the Monte Carlo sampling strategy

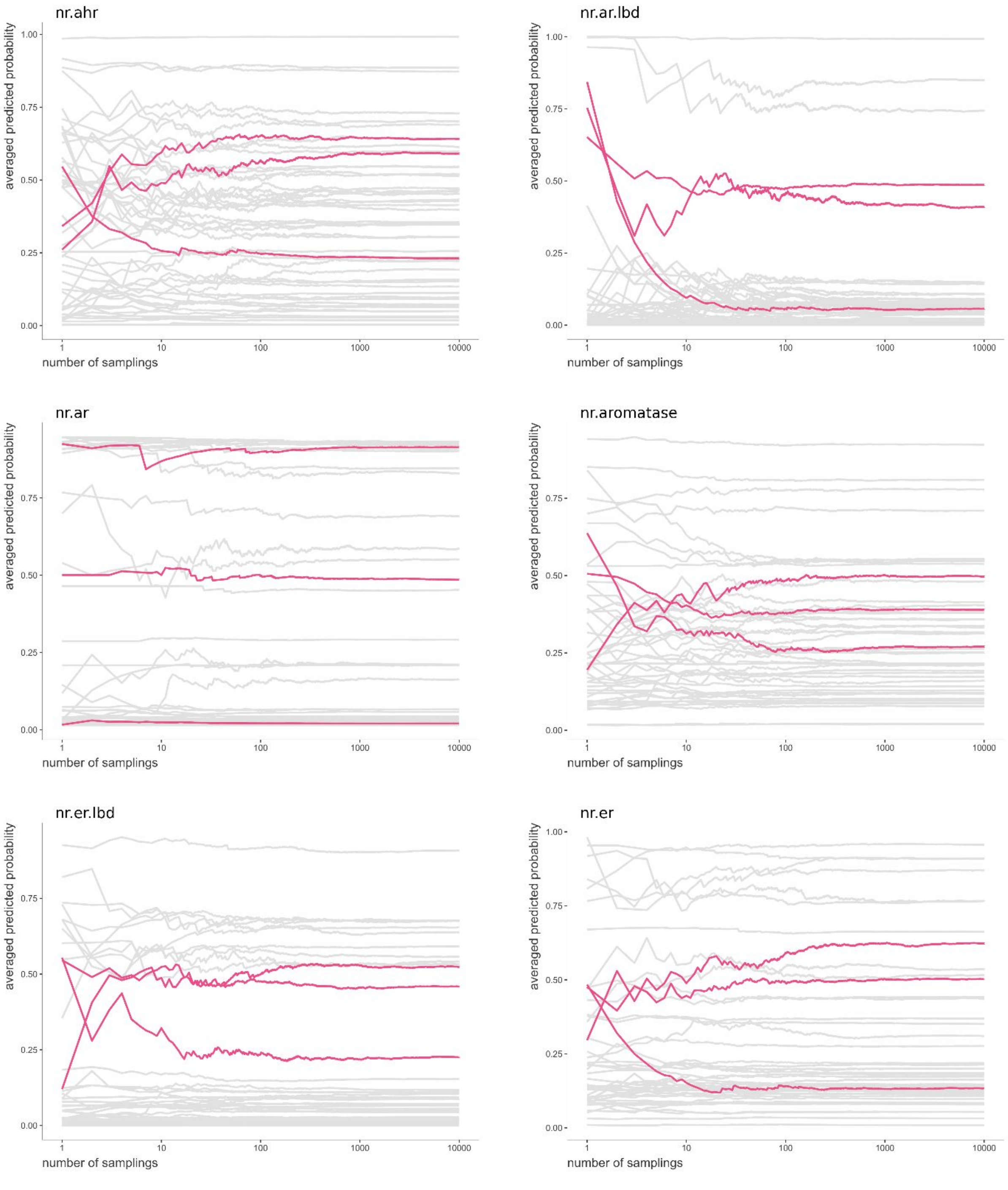

Figure S15. The effect of different sampling iterations (used in the conversion of SIRIUS+CSI:FingerID outputted fingerprint features to the true binary fingerprint features) on the predictions of the single-output model for 50 chemicals in the six bioassays. Each line represents the predictions made for one chemical, and the pink lines illustrate the importance of adequate sampling iterations. This figure is reprinted from[1].

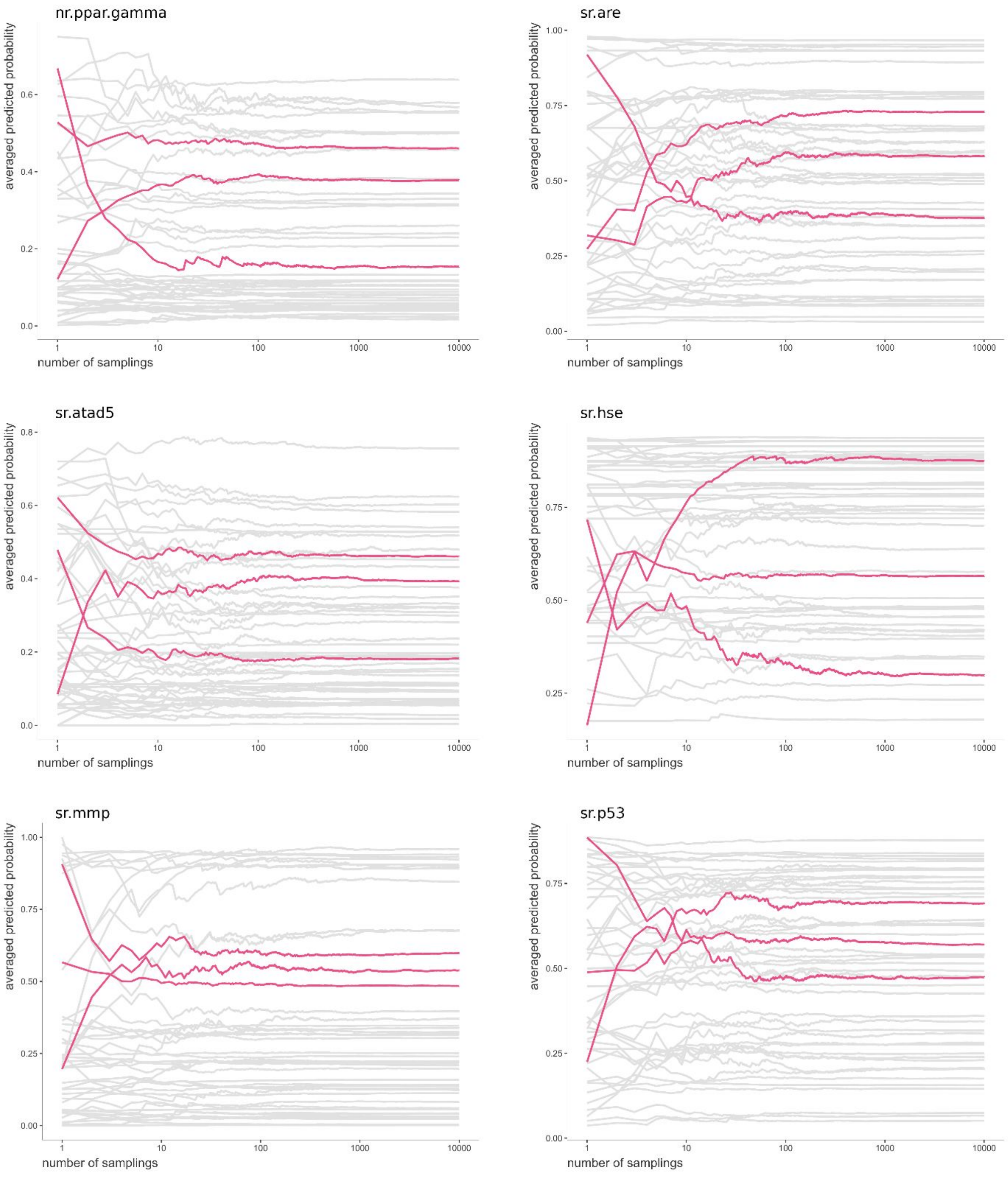

Figure S16. The effect of different sampling iterations (used in the conversion of SIRIUS+CSI:FingerID outputted fingerprint features to the true binary fingerprint features) on the predictions of the single-output model for 50 chemicals in the six bioassays. Each line represents the predictions made for one chemical, and the pink lines illustrate the importance of adequate sampling iterations. This figure is reprinted from[1].

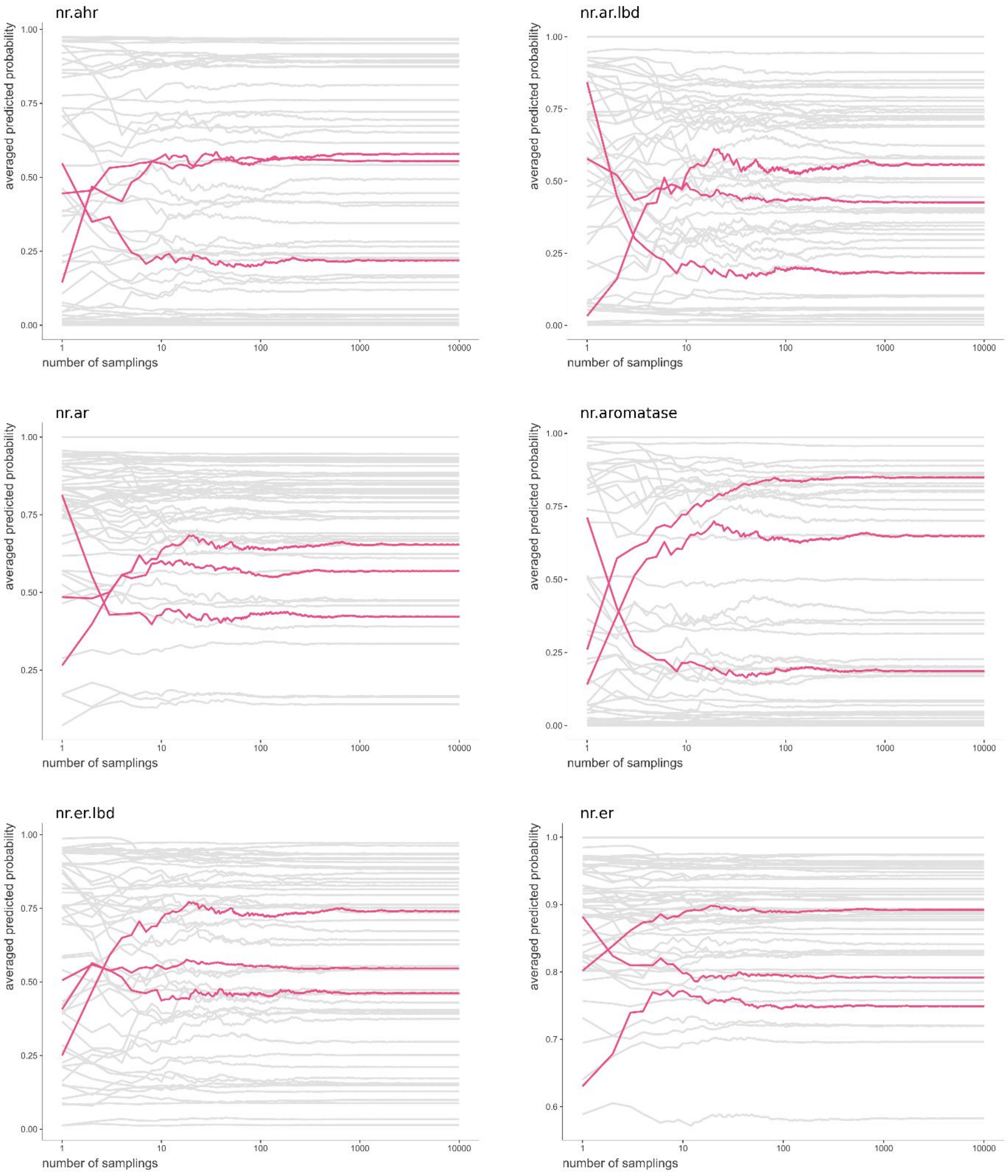

Figure S17. The effect of different sampling iterations (used in the conversion of SIRIUS+CSI:FingerID outputted fingerprint features to the true binary fingerprint features) on the predictions of the multi-output model for 50 chemicals in the six bioassays. Each line represents the predictions made for one chemical, and the pink lines illustrate the importance of adequate sampling iterations. This figure is reprinted from[1].

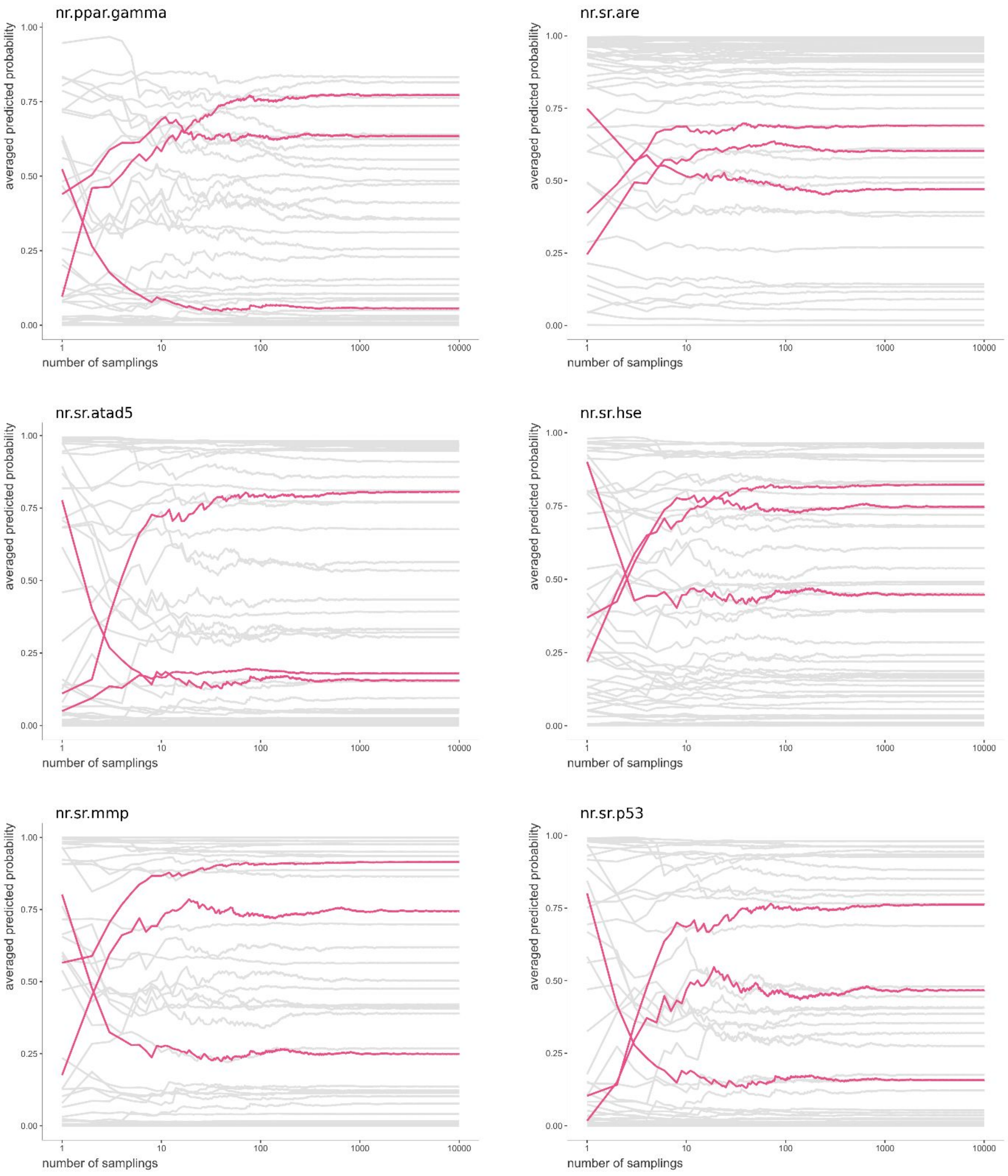

Figure S18. The effect of different sampling iterations (used in the conversion of SIRIUS+CSI:FingerID outputted fingerprint features to the true binary fingerprint features) on the predictions of the multi-output model for 50 chemicals in the six bioassays. Each line represents the predictions made for one chemical, and the pink lines illustrate the importance of adequate sampling iterations. This figure is reprinted from[1].

Table S9. The number of chemicals per bioassay, which predicted endocrine-disrupting activity, would have produced a different result after the first iteration compared to the 10,000th iteration. This table is reprinted from[1].

bioassay	number of chemicals	
	single-output model	multi-output model
nr.ahr	61	35
nr.ar.lbd	12	60
nr.ar	1	40
nr.aromatase	30	35
nr.er.lbd	7	59
nr.er	14	0
nr.pppar.gamma	6	31
sr.are	59	13
sr.atad5	17	62
sr.hse	47	55
sr.mmp	17	41
sr.p53	52	59

Table S10. Performance of the models on the real-life test set, calculated using the naive approach with a threshold value of 0.5 to enable the use of probabilistic fingerprint features

bioassay	$FPR_{TPR = 0.9}$	
	single-output models	multi-output model
nr.ahr	0.514	1.000
nr.ar.lbd	1.000	1.000
nr.ar	0.919	1.000
nr.aromatase	0.478	1.000
nr.er.lbd	0.794	1.000
nr.er	0.842	0.882
nr.pppar.gamma	0.616	1.000
sr.are	0.674	0.866
sr.atad5	0.519	1.000
sr.hse	0.807	1.000
sr.mmp	0.217	1.000
sr.p53	0.537	0.395

Implementing the Monte Carlo sampling strategy, while enhancing prediction accuracy, does entail additional computational time compared to a simple threshold-based approach. Specifically, predicting the activity of a single chemical requires approximately 3 minutes on a single CPU (AMD Ryzen Threadripper PRO 5955WX 16-Cores 4.00 GHz). It is important to note that the computational time does not scale linearly with the number of chemicals being predicted. For instance, predicting the activity of 10 chemicals takes around 5 minutes, while for 100 chemicals, it increases to approximately 36 minutes, and for 250 chemicals, it is approximately 86 minutes. These computational times align with typical processing times for nontarget LC/ESI/HRMS data analysis, where raw data processing usually takes several tens of hours.

Section S9: Effect of the usage of SIRIUS+CSI:FingerID on the applicability of models

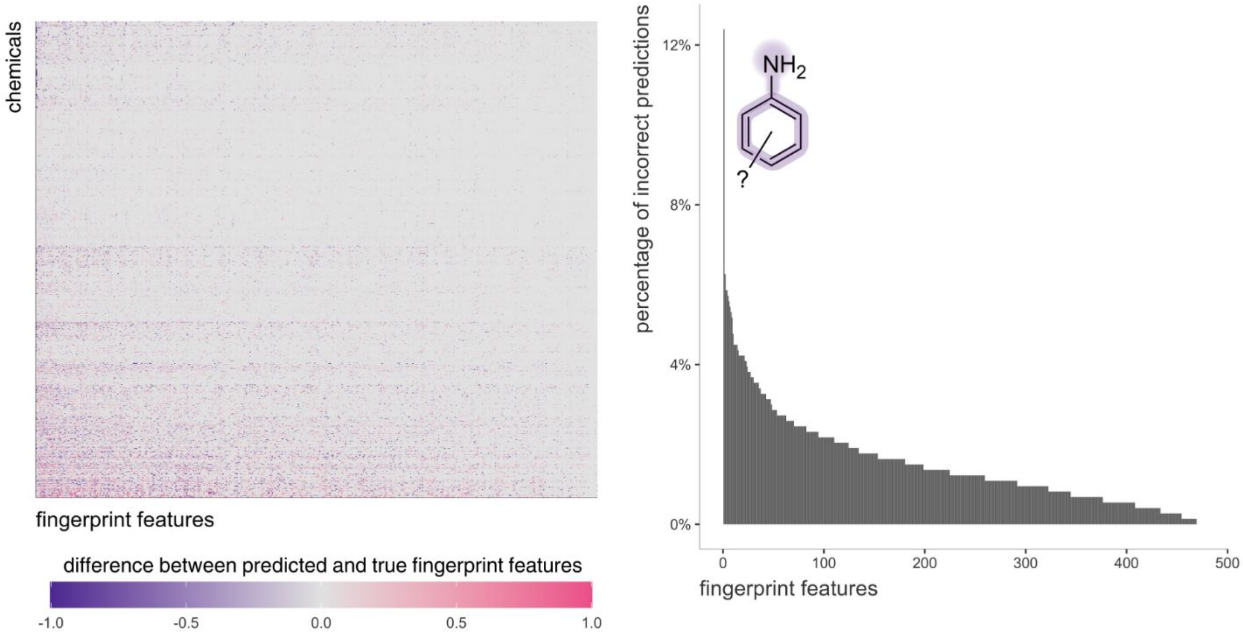

Figure S19. Difference between the predicted fingerprint features, calculated based on HRMS data using SIRIUS+CSI:FingerID ($fpf_{\text{predicted}}$; posterior probabilities) and the true fingerprint features derived from SMILES (fpf_{true}). The figure includes data on 476 molecular properties selected for model training, which are arranged in the same order on both panels. The left panel depicts the difference between $fpf_{\text{predicted}}$ and fpf_{true} for each chemical in the real-life test set, calculated as $difference = fpf_{\text{predicted}} - fpf_{\text{true}}$. The right panel illustrates the proportions of chemicals for which the predicted and true fingerprint features disagree. For that, a naive approach was used: setting a feature value to "1" if $fpf_{\text{predicted}} \geq 0.5$ and "0" otherwise, and then comparing the results with true values. This figure is reprinted from[1].

References

(1) Rahu, I. Machine Learning for Assessing Toxicity of Chemicals Identified with Mass Spectrometry, University of Tartu, 2023. https://comserv.cs.ut.ee/ati_thesis/datasheet.php?id=77585&language=en (accessed 2023-10-31).

(2) Chawla, N. V.; Bowyer, K. W.; Hall, L. O.; Kegelmeyer, W. P. SMOTE: Synthetic Minority Over-Sampling Technique. *J. Artif. Intell. Res.* **2002**, *16*, 321–357. https://doi.org/10.1613/jair.953.

(3) Lunardon, N.; Menardi, G.; Torelli, N. ROSE: A Package for Binary Imbalanced Learning. *R J.* **2014**, *6*, 79. https://doi.org/10.32614/RJ-2014-008.
